# Low nephron endowment increases susceptibility to renal stress and chronic kidney disease

**DOI:** 10.1172/jci.insight.161316

**Published:** 2023-02-08

**Authors:** Pamela I. Good, Ling Li, Holly A. Hurst, Ileana Serrano Herrera, Katherine Xu, Meenakshi Rao, David A. Bateman, Qais Al-Awqati, Vivette D. D’Agati, Frank Costantini, Fangming Lin

**Affiliations:** 1 Department of Pediatrics and; 2 Department of Internal Medicine, Columbia University Vagelos College of Physicians and Surgeons New York, New York, USA.; 3 Department of Pediatrics, Boston Children’s Hospital and Harvard Medical School, Boston Massachusetts, USA.; 4 Department of Pathology and Cellular Biology at Columbia University Vagelos College of Physicians and Surgeons, New York, New York, USA.; 5 Department of Genetics and Development at Columbia University Vagelos College of Physicians and Surgeons, New York, New York, USA.

**Keywords:** Nephrology, Chronic kidney disease

## Abstract

Preterm birth results in low nephron endowment and increased risk of acute kidney injury (AKI) and chronic kidney disease (CKD). To understand the pathogenesis of AKI and CKD in preterm humans, we generated potentially novel mouse models with a 30%–70% reduction in nephron number by inhibiting or deleting Ret tyrosine kinase in the developing ureteric bud. These mice developed glomerular and tubular hypertrophy, followed by the transition to CKD, recapitulating the renal pathological changes seen in humans born preterm. We injected neonatal mice with gentamicin, a ubiquitous nephrotoxic exposure in preterm infants, and detected more severe proximal tubular injury in mice with low nephron number compared with controls with normal nephron number. Mice with low nephron number had reduced proliferative repair with more rapid development of CKD. Furthermore, mice had more profound inflammation with highly elevated levels of MCP-1 and CXCL10, produced in part by damaged proximal tubules. Our study directly links low nephron endowment with postnatal renal hypertrophy, which in this model is maladaptive and results in CKD. Underdeveloped kidneys are more susceptible to gentamicin-induced AKI, suggesting that AKI in the setting of low nephron number is more severe and further increases the risk of CKD in this vulnerable population.

## Graphical abstract

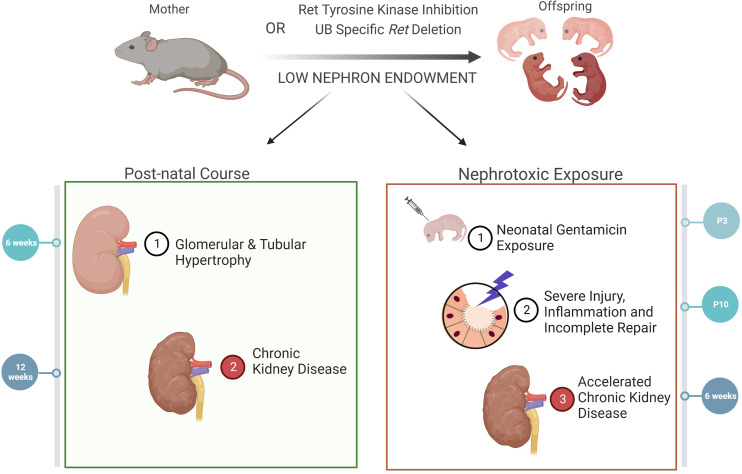

## Introduction

The global rate of preterm birth (birth before 37 weeks gestation) is roughly 11%, with 15 million premature births each year (1, 2). With advances in neonatal care, more preterm infants are surviving to adulthood, albeit with long-term health consequences (3). Preterm birth is a risk factor for acute kidney injury (AKI), which is associated with increased morbidity and mortality in the neonatal period (4). AKI increases the risk for developing CKD (5, 6), and survivors of prematurity have increased incidence of chronic kidney disease (CKD) later in life (7–9). Nephrogenesis is not complete until 34–36 weeks gestation, and 60% of nephrons are formed during the third trimester (10). Human autopsy studies show that, at birth, preterm infants have reduced nephron number. Studies have also suggested that, while there may be a limited period of postnatal nephrogenesis, a significant number of glomeruli appear abnormal, suggesting that ex utero nephrogenesis is perturbed (11, 12). This results in low functional nephron mass in humans born preterm, particularly those born extremely preterm (birth before 28 weeks gestation).

Brenner et al. were the first to hypothesize that low nephron endowment in humans increases an individual’s risk for hypertension and CKD later in life (13–17). Using an adult rat model of 5/6 nephrectomy, Brenner and others showed that remnant nephrons underwent hyperfiltration to increase the single-nephron glomerular filtration rate (GFR). These altered hemodynamics led to maladaptive changes resulting in eventual glomerular sclerosis, nephron drop out, and renal failure (18, 19). Less drastic reduction in nephron number, by removing 1 kidney, does not result in progressive renal failure; otherwise, kidney donation for transplantation would not be acceptable. However, studies in adult rodents do not recapitulate the reduced nephron mass seen in preterm humans because, in adults, hyperfiltration occurs abruptly after surgical reduction of nephron mass and not during the critical period of postnatal renal growth and maturation. Several models of congenitally reduced nephron number exist. One model uses restriction in maternal protein and calorie intake during gestation (20–23); however, this leads to generalized epigenetic responses and affects multiple organs in offspring (24–27). Another model resembling premature kidneys involves cesarean delivery 1–2 days prior to natural birth in mice and results in reduced nephron endowment with evidence of CKD manifested by albuminuria, hypertension, and lower GFR 5 weeks later. While this study recapitulates human premature birth, the nephron deficits are mild (20%), and this likely resembles a late preterm human gestation (28).

To understand the pathogenesis of AKI and CKD in the growing population of humans born preterm, we generated potentially novel mouse models of congenitally low nephron number. Since glial cell–derived neurotrophic growth factor (GDNF)/Ret signaling plays a critical role in ureteric bud (UB) branching morphogenesis and nephron induction, we used chemical or genetic approaches to manipulate Ret tyrosine kinase expression or activity during the late stage of kidney development. We generated a cohort of mice with 30%–70% reduction in nephron number. These models recapitulate the likely spectrum of nephron endowment at birth in humans born preterm (10). We showed that mice with congenitally reduced nephron number undergo postnatal compensatory glomerular and tubular hypertrophy. By 6–12 weeks, they begin to show signs of developing CKD. Neonatal mice with low nephron number are more susceptible to gentamicin-induced AKI, with more severe injury and a unique and exaggerated inflammatory response originating in damaged proximal tubular cells. There is incomplete tubular repair and the accelerated emergence of a CKD phenotype. Overall, our study shows that mice with low nephron number that simulate preterm human kidneys develop compensatory hypertrophy, which becomes maladaptive with resultant CKD even in the absence of prior AKI. Additionally, these kidneys are at high risk of AKI, highlighting the vulnerability of underdeveloped kidneys.

## Results

### Inhibition or deletion of ret tyrosine kinase reduces nephron number.

**Figure 1:**
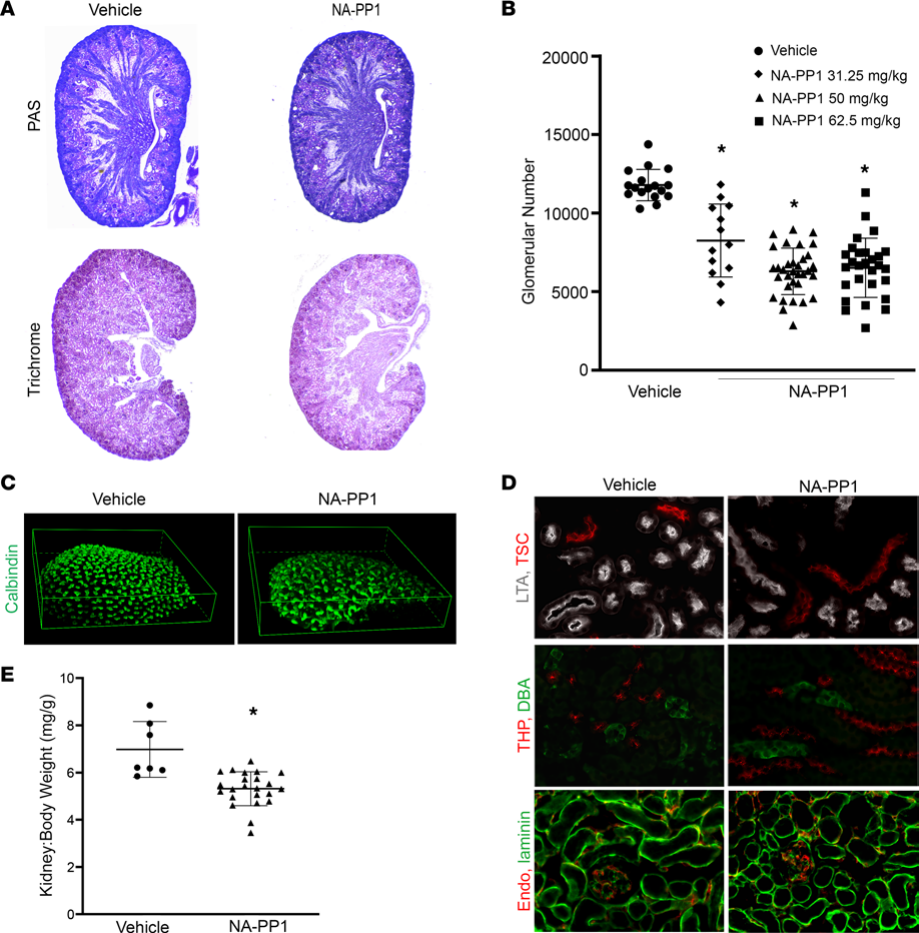
Inhibition of Ret tyrosine kinase resulted in reduced N^glom^. Pregnant Ret^flox-V805A^ mice were injected daily with vehicle or a small-molecule Ret tyrosine kinase inhibitor, NA-PP1, beginning E16.5 daily for 3 days. Renal structure and N^glom^ in offspring were analyzed. (**A**) PAS staining of P1 kidneys exposed to 50 mg/kg of NA-PP1 shows that kidneys are smaller than vehicle-exposed controls and have no tubular dilatation or hydronephrosis; images were obtained with a Zeiss M2 Bio microscope with 10× eyepiece and mechanical stage adjusted to a total of 33× magnification. Trichrome staining of P1 kidneys indicates no evidence of fibrosis (magnification, 25×). (**B**) N^glom^ was reduced in mice with prenatal exposure to NA-PP1 (32.25 mg/kg, *n* = 13; 50 mg/kg, *n* = 33; or 62.5 mg/kg, *n* = 28) compared with vehicle-exposed controls (*n* = 17). *P < 0.0001, 1-way ANOVA followed by Tukey’s test for multiple comparisons; all groups significantly different from vehicle. (**C**) NA-PP1–exposed mice show decreased UB branching and truncated UB tips. Whole-mount P1 kidneys from vehicle- or NA-PP1–exposed (50 mg/kg) pups were immunolabeled with antibody to calbindin, followed by optical clearing before image acquisition with a laser confocal microscope and 3-D reconstruction (100× magnification). (**D**) Two-week-old kidneys of mice with prenatal exposure to NA-PP1 (50 mg/kg) contain proximal tubules, thick ascending limbs, distal convoluted tubules, and glomerular and peritubular capillaries. Kidneys were stained with proximal tubule marker LTA (white) and distal tubule marker TSC (red), shown in top panel. Thick ascending limb is labeled with THP (red) and collecting ducts with DBA (green) shown in middle panel. Bottom panel shows capillaries and small veins labeled with endomucin (red). Laminin immunostaining (green) highlights tubular basement membrane (400× magnification). (**E**) Kidney/body weight ratio in NA-PP1–exposed (50 mg/kg) adult mice is significantly lower than age-matched vehicle-exposed controls. *P < 0.01, Welch’s *t* test, vehicle *n* = 7; NA-PP1, *n* = 23.

Nephrogenesis depends on reciprocal interaction between the UB and metanephric mesenchyme (MM). In the embryonic kidney, *Ret* is expressed in the UB, and its ligand, GDNF, is secreted by the surrounding MM. This interaction results in UB branching, which induces cells in the MM to condense around UB tips, transition to renal progenitor cells, and ultimately form the nephron (29–33). Genetic manipulations such as noninducible knocking-out of *Ret* or *GDNF* in mice results in renal agenesis or severe hypodysplasia (34, 35), limiting our ability to use these tools to generate mice with low nephron number and simulate renal consequences of human preterm birth. A mouse line with a floxed *Ret* allele that is engineered with a single amino acid substitution (V805A) at the ATP binding site of the Ret protein has no discernable abnormalities at the baseline (36, 37) but increases the receptor’s sensitivity to a small-molecule ATP competitive inhibitor, NA-PP1, by approximately 1,000-fold over WT tyrosine kinases (IC_50_ of nM versus μM) (38–41). We refer to this strain as *Ret^flox-V805A^*. We injected pregnant females with vehicle or NA-PP1 at 32.25 mg/kg, 50 mg/kg, or 62.5 mg/kg once a day i.p. starting E16.5 for 3 consecutive days. The small-molecule inhibitor–based approach generated viable offspring. Examination of more than 85 mice showed no evidence of hydronephrosis (Figure 1A), suggesting no obstruction of the collecting system and lower urinary tract. Although kidneys exposed to NA-PP1 were smaller at birth, Masson’s trichrome staining revealed no fibrosis (Figure 1A). While glomerular number (N^glom^), quantified using established acid maceration methods (42), was 11,790 ± 998.9 per kidney in vehicle-treated mice, exposing offspring to NA-PP1 in utero resulted in 30%–50% reduction of N^glom^ with a mean glomerular number of 8,250 ± 2,323; 6,293 ± 1,479; and 6,520 ± 1,884 in pups exposed to 32.25 mg/kg, 50 mg/kg, or 62.5 mg/kg, respectively. All doses of NA-PP1 yielded a significant reduction in N^glom^ compared with vehicle-exposed controls (*n* = 13–33 per group, *P* < 0.001; Figure 1B). To confirm that Ret activity was decreased, we performed immunoblot analyses using an anti-Ret antibody that recognizes phospho-Ret (upper band,175 kb) and Ret protein (lower band,155 kb) in E17.5 kidney homogenates from mice exposed to NA-PP1 or vehicle. The results show that kidneys exposed to NA-PP1 had reduced phospho-Ret as well as Ret proteins, which reflects that inhibition of Ret tyrosine kinase activity results in fewer Ret-containing UB tips (Supplemental Figure 1; supplemental material available online with this article; https://doi.org/10.1172/jci.insight.161316DS1).

We focused on analysis of mice born to mothers treated with 50 mg/kg of NA-PP1 (lowest dose that resulted in roughly 50% nephron reduction). Whole-mount staining with calbindin followed by 3-D image reconstruction of kidneys on day of birth (P1) showed reduced UB branching with truncated UB tips (Figure 1C), suggesting that Ret tyrosine kinase inhibition reduced nephron induction. Immunostaining of 2-week-old kidneys exposed to NA-PP1 in utero (50 mg/kg) revealed expression for markers of proximal tubules (Lotus Tetragonolobus Lectin [LTA]), thick ascending limb (Tamm-Horsfall protein [THP]), distal tubules (thiazide-sensitive Na-Cl cotransporter [TSC]), and collecting ducts (Dolichos biflorus agglutinin [DBA]). All proximal tubules contained brush border and stained positive for LTA. While there appeared to be less LTA fluorescence signals in NA-PP1–exposed sections, this is likely due to nephron number deficits. NA-PP1–exposed mice had patent glomerular capillary tufts and peritubular capillaries (Figure 1D). These results indicate that inhibiting Ret tyrosine kinase activity with 50 mg/kg of NA-PP1 beginning E16.5 did not prevent formation of these renal structural components. Kidney/body weight ratio was significantly lower in mice exposed to NA-PP1 compared with vehicle-exposed mice (Figure 1E).

Because Ret signaling is required for neural crest cell migration to the gut mesenchyme (from E9 to E14) (43, 44) and may play a role in the enteric neuron survival (36), we examined distal colons for the number and organization of neurons. Treatment on or after E16.5 resulted in no significant differences in the number of neurons in the distal colon. The neuronal organization was similar to vehicle-exposed controls (Supplemental Figure 1). NA-PP1–exposed mice had adequate postnatal weight gain, suggesting sufficient gastrointestinal function.

**Figure 2:**
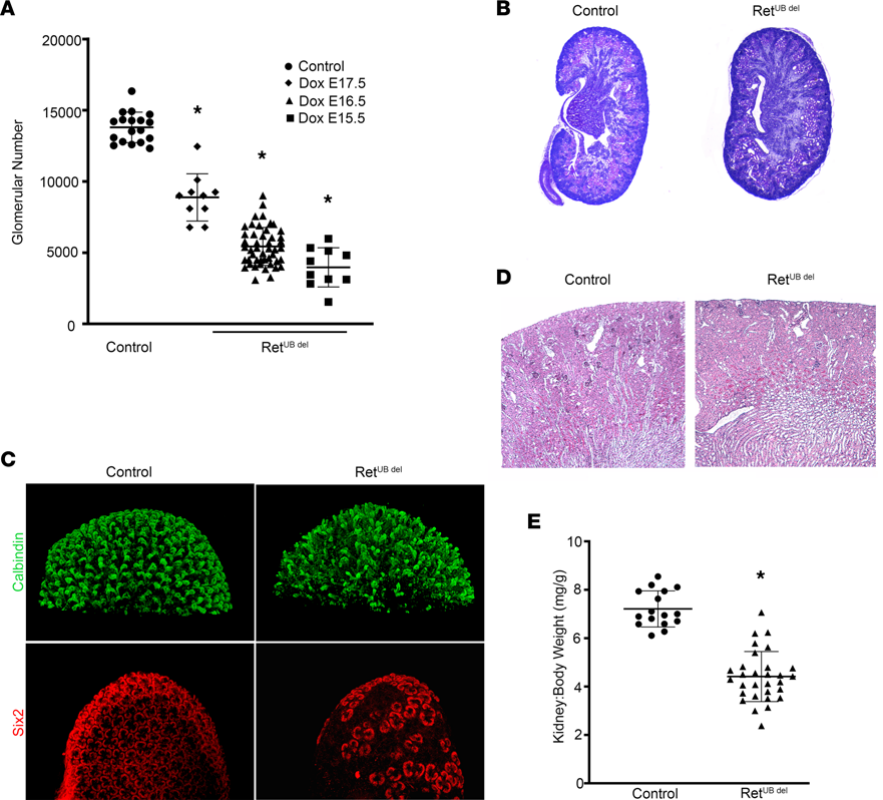
*Ret* deletion results in decreased N^glom^ in a time-dependent manner. Pregnant female mice carrying the *Hoxb7-rtTA;tet-O-Cre;Ret^flox-V805A^* transgenes were crossed with male mice of the same genotype and exposed to doxycycline (Dox) in the drinking water beginning E15.5, E16.5, or E17.5 through delivery. Offspring carrying all transgenes are named Ret^UB^ ^del^, while littermates carrying *tet-O-Cre; Ret^flox-V805A^* without *Hoxb7rtTA* are controls. (**A**) UB-specific deletion of *Ret* during kidney development leads to significant reduction of N^glom^ with greater reduction on earlier exposure (71% at E15.5, *n* = 10; 60% at E16.5, *n* = 47; and 36% at E17.5, *n* = 10; compared with controls, *n* = 18). *P < 0.0001, 1-way ANOVA followed by Tukey’s test for multiple comparisons; all groups significantly differed from controls. (**B**) PAS staining of P1 kidneys from Ret^UB^ ^del^ pups exposed to Dox E16.5 shows no tubular dilatation or hydronephrosis (imaged at 33×). (**C**) *Ret* deletion (starting E16.5) resulted in decreased UB branching and truncated UB tips with areas of absent Six2-expressing cap mesenchyme. Whole-mount P1 kidneys were labeled with antibody to calbindin to identify UB and its derivatives (green) or antibody to Six2 to identify nephron progenitors in the cap mesenchyme (red), followed by optical clearing prior to confocal microscopy and 3-D image reconstruction (100× magnification). (**D**) PAS staining of adult Ret^UB^ ^del^ mouse kidneys (6 weeks old) shows thinner cortex with decreased glomerular number (40× magnification). (**E**) Adult kidney/body weight ratio in Ret^UB^ ^del^ mice (Dox E16.5) is significantly lower than age-matched littermate controls. *P < 0.001, Welch’s *t* test with *n* = 16 controls and *n* = 30 Ret^UB^ ^del^ mice.

To circumvent potential effects of systemic Ret tyrosine kinase inhibition, we deleted *Ret* specifically in the developing kidney by taking advantage of the UB-specific expression of *Hoxb7-rtTA* combined with the *tet-O-Cre* system (35, 45, 46). Since the engineered *Ret* allele *(Ret^flox-V805A^*) is floxed, treating pregnant *Hoxb7-rtTA;tet-O-Cre;Ret^flox-V805A^* mice with doxycycline (Dox) is expected to delete *Ret* in the UB of offspring carrying the same transgenes (named Ret^UB^ ^del^ mice), thus reducing UB branching and decreasing nephron induction. Our breeding strategy yielded littermate controls with the genotype of *tet-O-Cre;Ret^flox-V805A^* lacking *rtTA* and unable to activate *tet-O-Cre*. Previous work shows that *HoxB7-rtTA* mice expressed high levels of rtTA and that induction of Cre activity is achieved after 1-day treatment with Dox in the drinking water (45). We treated pregnant females with Dox beginning at E15.5, E16.5, or E17.5 through delivery. Analysis of adult offspring kidneys showed significant reduction of N^glom^ to 3,977 ± 1,377; 5,440 ± 1,333; or 8,888 ± 1,657 with Dox treatment beginning E15.5, E16.5, or E17.5, respectively, compared with 13,800 ± 1,069 in littermate controls (*n* = 10–47/group, *P* < 0.05 for all comparisons) (Figure 2A). No hydronephrosis was observed with *Ret* deletion on E16.5 (Figure 2B). Kidneys contained patent glomerular capillary tufts and peritubular capillaries and expressed markers for proximal tubules, thick ascending limbs, distal tubules, and collecting ducts (Supplemental Figure 2). Furthermore, there were no morphological changes suggestive of renal dysplasia, supporting the utility of these mice as a model of congenitally low nephron endowment.

We chose mice exposed to Dox starting E16.5 for detailed analysis. First, we detected no differences in the mean glomerular number between control females (average N^glom^ = 13,594) and control males (average N^glom^ = 14,212, *P* = 0.35, *n* = 6–12). There were no sex-related differences in the mean glomerular number in Ret^UB^ ^del^ mice, with the average N^glom^ of 5,522 in females and average N^glom^ of 5,338 in males (*P* = 0.65, *n* = 21–26). At P1, whole-mount kidneys from mice exposed to Dox staring at E16.5 showed truncated UB branching with qualitatively decreased UB tips and reduced Six2-expressing cap mesenchyme (Figure 2C), suggesting that reduced GDNF/Ret signaling in the developing kidney attenuated UB branching and nephron induction. The kidneys had fewer glomeruli (Figure 2D) and were smaller, with significantly reduced kidney/body weight ratios compared with controls (Figure 2E). No interstitial fibrosis was identified at early time points (up to P10; data not shown). This cohort of Ret^UB^ ^del^ mice provided a range of 35%–70% nephron reduction, simulating underdeveloped kidneys in humans born preterm (47).

### Mice with low nephron number develop glomerular and tubular hypertrophy.

**Figure 3:**
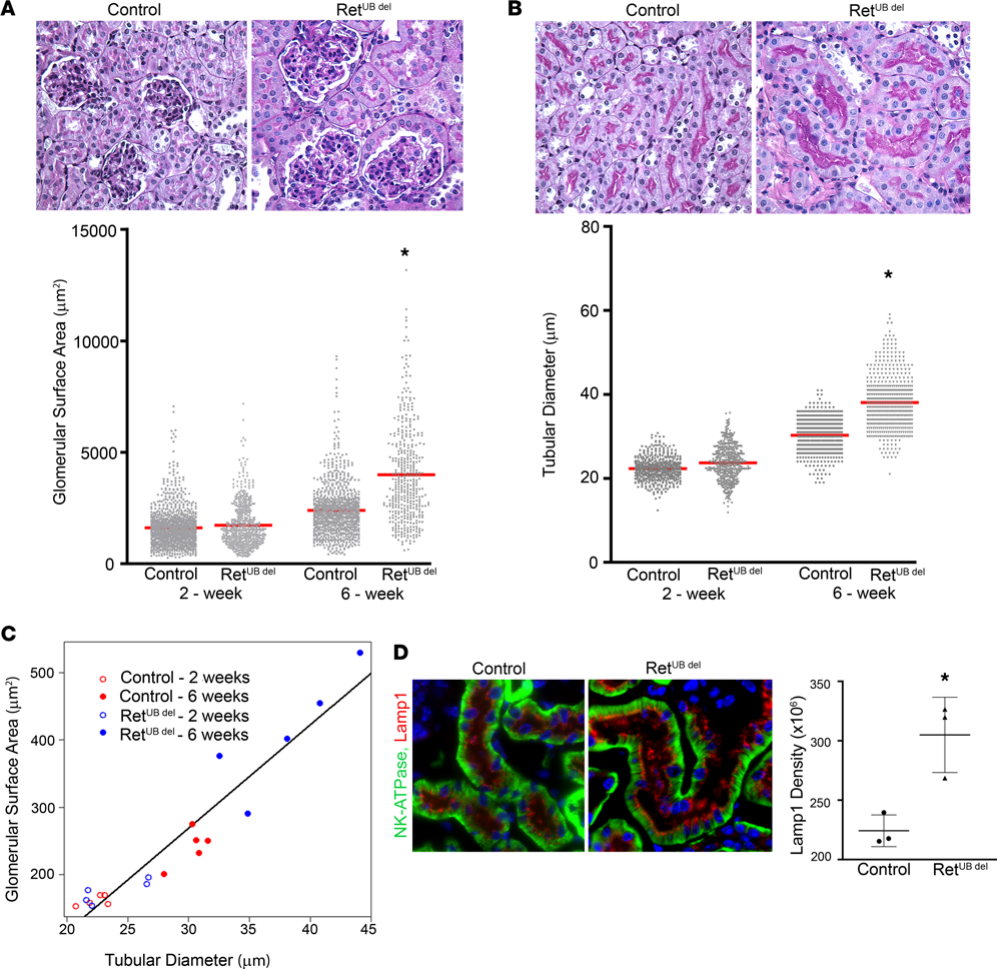
Ret^UB^ ^del^ mice develop glomerular and tubular hypertrophy. (**A**) Top panel, PAS-stained kidneys at 6 weeks of age show glomerular hypertrophy (imaged at 400×) in Ret^UB^ ^del^ mice. Bottom panel, quantification of glomerular surface area at 2 and 6 weeks of age. While at 2 weeks there was no difference between groups (mixed-effects regression; mice, *n* = 10; glomeruli, *n* = 1,741; *P* = 0.61), by 6 weeks, Ret^UB^ ^del^ mice developed glomerular enlargement, with mean glomerular surface area 1,332 μm^2^ larger in Ret^UB^ ^del^ compared with controls (mixed-effects regression; mice, *n* = 10; glomeruli, *n* = 1,351; *P* = 0.002). (**B**) Top panel, PAS-stained kidneys at 6 weeks of age show tubular hypertrophy (imaged at 400×) in Ret^UB^ ^del^ mice. Bottom panel, quantification of proximal tubular diameter (measured in the S3 segments) at 2 and 6 weeks of age. While, at 2 weeks, there was no difference between groups (mixed-effects regression; mice, *n* = 10; tubules, *n* = 1,000; *P* = 0.317), by 6 weeks, Ret^UB^ ^del^ mice developed tubular enlargement, with mean tubular diameter 7.8 μm larger in Ret^UB^ ^del^ compared with controls (mixed-effects regression; mice, *n* = 10; tubules, *n* = 1,000; *P* = 0.007). (**C**) Correlation of glomerular and tubular size with tubular diameter plotted against glomerular surface area showed that mean tubular diameter and mean glomerular surface area are highly correlated; *R* = 0.917. (**D**) Ret^UB^ ^del^ mice at 6 weeks of age have robust endolysosomal structures. Lamp1 labels exuberant and abundant late endosomes and lysosomes in Ret^UB^ ^del^ mice. Immunolabeling of N^+^/K^+^-ATPase highlights basolateral cell membranes (400× magnification). Quantification of Lamp1 fluorescence integrated density corrected for number of nuclei in proximal tubules localized to the cortex shows a significant increase in Lamp1 expression at 6 weeks of age in Ret ^UB^ ^del^ mice. *P = 0.03 using Welch’s *t* test; *n* = 3 for each group.

Given the increased workload of individual nephrons in mice with congenitally low nephron number, we next assessed for postnatal renal adaptation to low nephron endowment. We chose kidneys exposed to Dox starting E16.5 (40%–60% reduction of N^glom^) for quantification of glomerular size and tubular diameter over the course of renal growth and maturation. While no significant differences were detected between Ret^UB^ ^del^ mice and controls at 2 weeks of age (5 mean difference of 9.4 µm^2^; 95% CI, –150.2 to 303.0; *P* = 0.61), Ret^UB^ ^del^ mice developed glomerular enlargement over the course of next 4 weeks (Figure 3A). By 6 weeks of age, the mean glomerular surface area in Ret^UB^ ^del^ mice was 1,332 μm^2^ (95% CI, 6,72.9–2,140.7 μm^2^) or 61.1% (95% CI, 30.9–98.3 µm^2^) larger than that of controls (*P* = 0.002). While glomerular surface area increased between 2 and 6 weeks in both groups, it increased at an accelerated rate in mice with low nephron number, and the difference in growth trajectories was significant (excess growth of 1,272.49 μm^2^; 95% CI, 1,000.1–1,606.9 µm^2^; *P* = 0.0011).

Tubular hypertrophy also occurred between 2 and 6 weeks of age (Figure 3B). Since studies in hypertrophic kidneys of experimental animals indicate that the most prominent tubular hypertrophy occurs in the proximal tubules (48, 49), we measured tubular diameter in the S3 segment (pars recta) of proximal tubules, localized to the outer stripe of the outer medulla. At 2 weeks, there were no differences between Ret^UB^ ^del^ and controls (1.37 μm; 95% CI, –1.15 to 3.88 µm; *P* = 0.317) (Figure 3B); however, by 6 weeks, the mean diameter in Ret^UB^ ^del^ mice was 7.81 μm (95% CI, 3.61–12.01 µm) larger than that of controls (*P* = 0.007). While proximal tubular diameter increased in both groups between 2 and 6 weeks of age, the increase was greater in the Ret^UB^ ^del^ group (excess growth of 6.44 μm; 95% CI, 1.85–11.04 µm; *P* = 0.02). Interestingly, glomerular and tubular size were highly correlated (Figure 3C) with a correlation coefficient of *R* = 0.917 (95% CI, 0.680–0.981). The degree of glomerular hypertrophy is paralleled in the proximal tubules; more hypertrophy in both nephron components was expected in mice with the lowest nephron number within the Ret^UB^ ^del^ group, suggesting that glomerular hyperfiltration and tubular hypertrophy are interrelated.

Proximal tubular brush border appeared exuberant in Ret^UB^ ^del^ mice as early as 2 weeks of age. At 6 weeks, immunostaining of NK-ATPase showed more elaborate basolateral membrane in-folding, suggesting a possible increase in epithelial transport activity. Immunostaining of Lamp1, a membrane protein located on late endosomes and lysosomes, revealed that Ret^UB^ ^del^ mice have a more robust endolysosomal system (Figure 3D), with increased integrated density of Lamp1 fluorescence signals (mean 224 × 10^3^ in controls versus mean 305 × 10^3^ in Ret^UB^ ^del^ mice; *P* = 0.03) per number of nuclei in the cortical region where S1 and S2 segments of the proximal tubule are located. These adaptive changes likely reflect increased glomerular and tubular function at the single-nephron level.

It is interesting to note that glomerular obsolescence with collapsed capillary loops were observed in the subcapsular region, where the newest generation of glomeruli are located (Supplemental Figure 3). This glomerular phenotype is reminiscent of human kidney autopsies showing that glomeruli in premature infants are structurally abnormal (11, 12). In contrast, the glomeruli located in the deeper cortex and corticomedullary junction were larger but morphologically similar to controls (data not shown).

Next, we tested whether 50% nephron reduction in adult mice resulted in similar glomerular and tubular adaptation. We performed right nephrectomy or sham operation in 8-week-old *Ret^flox-V805A^* mice (no NA-PP1 or Dox exposure) and analyzed kidneys 4 weeks later. We did not detect differences in the size of glomeruli. Although the tubular diameter was slightly larger in nephrectomized mice compared with sham-operated mice, the degree of hypertrophy was much less than that of mice with congenitally reduced nephron number (10% larger after nephrectomy versus 26% larger in mice with congenitally low nephron number). In addition, there was no evidence of interstitial fibrosis, inflammation, or tubular atrophy (Supplemental Figure 4).

Overall, our results suggest that our newly generated mouse models of low nephron endowment simulate postnatal renal compensatory hypertrophy in humans born with low nephron number, with hypertrophy and excess growth occurring between 2 and 6 weeks of age. Importantly, kidneys with congenital reduction of nephron number by 40%–60% had unique adaptive changes during the period of postnatal growth and maturation, whereas a similar nephron reduction in adults does not result in such adaptation.

### Adult Ret^UB^ ^del^ mice develop CKD.

**Figure 4:**
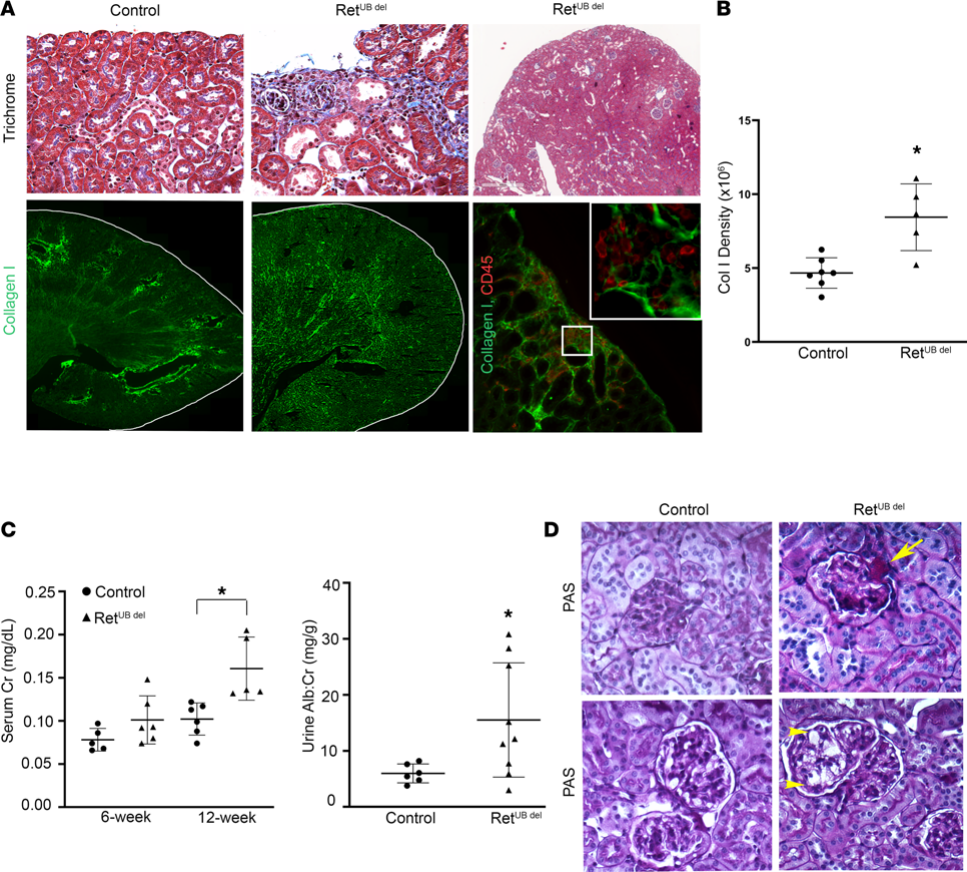
Adult Ret^UB^ ^del^ mice develop a CKD phenotype. (**A**) Top panel, trichrome staining shows focal subcapsular interstitial fibrosis and inflammation in Ret^UB^ ^del^ mouse kidneys at 6 weeks of age (400× magnification left 2 panels, 5.8× magnification right panel). Bottom panel left and middle, collagen I immunostaining shows increased collagen I expression in 6-week-old Ret^UB^ ^del^ mice compared with littermate control. Kidney images were contoured, tile scanned, and stitched into single mosaics (200× magnification) for collagen quantification. Bottom right, collagen I and CD45 staining show colocalization of areas of collagen I deposition and inflammatory cell infiltrates (200× magnification; inset 400× magnification) (**B**) Quantification of collagen I integrated density shows a significant increase in collagen I deposition at 6 weeks of age in Ret^UB^ ^del^ mice compared with controls (*P = 0.016, Welch’s *t* test, *n* = 7 controls, *n* = 5 Ret^UB^ ^del^ mice). (**C**) At 6 weeks of age sCr was similar in control and Ret^UB^ ^del^ mice (*P* = 0.13, Welch’s *t* test, *n* = 5 controls, *n* = 6 Ret^UB^ ^del^) with increased creatinine evident by 12 weeks of age (*P = 0.02, Welch’s *t* test, *n* = 6 control, *n* = 5 Ret^UB^ ^del^). By 12 weeks of age, there was increased urinary albumin excretion (*P = 0.02, Welch’s *t* test, *n* = 6 control, *n* = 9 Ret^UB^ ^del^). (**D**) PAS staining of kidneys at 9 months of age reveals perihilar hyalinosis (arrow) and segmental accumulation of endocapillary foam cells (arrowheads) forming a cellular lesion of segmental sclerosis (400× magnification).

Low nephron endowment predisposes humans to renal disease later in life (13, 16). In a rodent model of 5/6 nephrectomy (83% nephron reduction), hypertrophy and hyperfiltration at the single-nephron level alter renal hemodynamics, leading to maladaptive changes such as glomerular sclerosis, nephron dropout, and renal failure (18, 19). However, this occurs after the critical window of postnatal growth and maturation, and it may cause necrosis and inflammation at the incision margins. To test whether compensatory nephron hypertrophy due to congenital nephron deficits becomes maladaptive and contributes to the development of CKD, we performed serial examinations of Ret^UB^ ^del^ mice that were exposed to Dox (starting at E16.5) for up to 9 months. Fibrotic foci started to appear — especially in areas of glomerular obsolescence — at 6 weeks (Figure 4A), and there was significantly higher collagen I expression compared with age-matched controls (Figure 4, A and B). In areas of increased collagen I deposition, CD45^+^ inflammatory cells also accumulated as shown in Figure 4A. Tubular atrophy and interstitial inflammation emerged by 12 weeks (Supplemental Figure 3). Serum creatinine (sCr) was higher at 12 weeks (control 0.1 mg/dL versus Ret^UB^ ^del^ 0.16 mg/dL, *P* = 0.02, *n* = 11), and urinary albumin excretion increased (mean urine albumin/urine creatinine [Ualb:cr] 15.5 versus 5.98 mg/g in controls, *P* = 0.02, *n* = 15) (Figure 4C). By 9 months, glomerular changes secondary to hyperfiltration, which included perihilar hyalinosis and segmental luminal obliteration by endocapillary foam cells, appeared (Figure 4D). These changes have been shown to be early signs of developing focal segmental glomerular sclerosis (FSGS) (50).

In summary, we found that in mice with 30%–70% congenitally reduced nephron number, compensatory tubular and glomerular hypertrophy occurred between 2 and 6 weeks of age, and by 6–12 weeks of age, mice exhibit pathologic changes characteristic of CKD. This differs from adult mice with 50% reduced nephron number, as nephrectomized adults have no inflammation or morphologic changes resembling CKD. In mice with congenitally low nephron number, increased workload at the single-nephron level and compensatory hypertrophy appear to activate cellular stress and inflammatory pathways. The end point of this process is a steady decline in renal structure and function, even in the absence of additional stressors and injuries.

### Ret^UB^ ^del^ mice have more severe injury and accelerated development of CKD after neonatal AKI.

**Figure 5:**
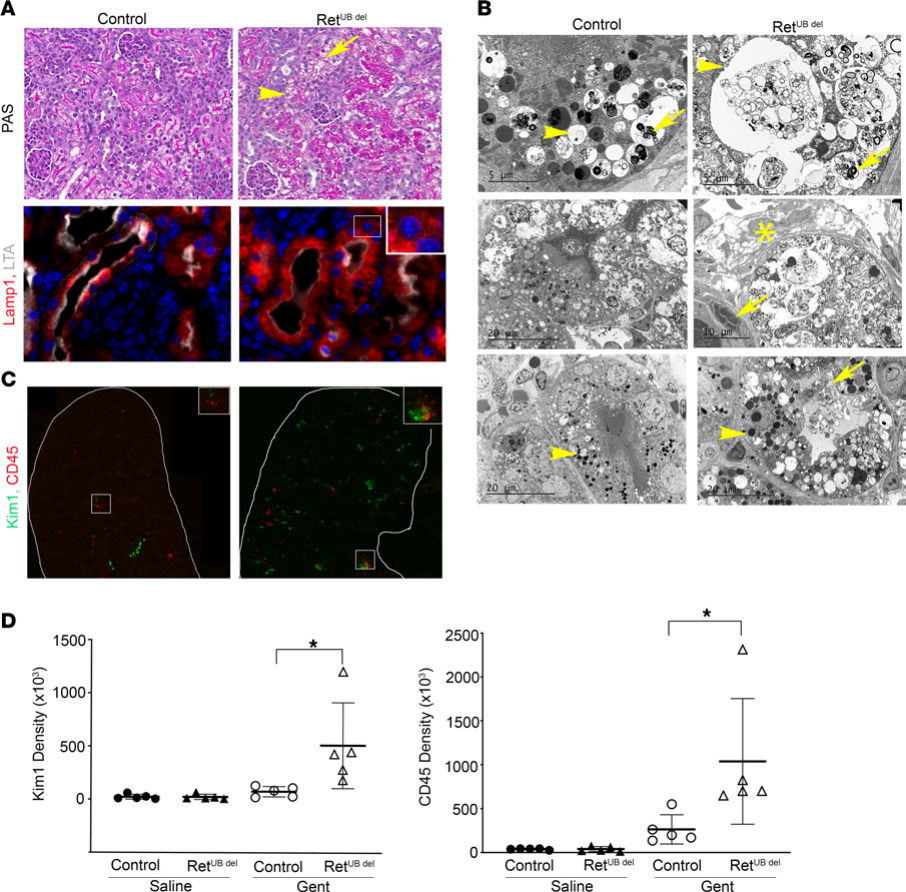
Neonatal Ret^UB^ ^del^ mice have more severe injury following gentamicin-induced AKI. Newborn mice (control and Ret^UB^ ^del^) were injected with saline or gentamicin once a day for 7 days from P3 to P9. Kidneys were collected for analysis 1 day after completing treatment (P10). (**A**) Top panel, PAS staining shows more tubular vacuolization (arrowhead) and loss of brush border in proximal tubules of Ret^UB^ ^del^ mouse kidneys (arrow). Bottom panel, more abundantly enlarged Lamp1-expressing lysosomes (red) in proximal tubules labeled with LTA (white) in Ret^UB^ ^del^ mice. Images were obtained at 630× magnification. Inset indicates swollen lysosomes (inset 1,260× magnification). (**B**) Cellular injury shown by electron microscopy. Top panels, lysosomes (arrowheads) containing myelin bodies (arrows) and cellular debris in proximal tubules of control and Ret^UB^ ^del^ mice. Middle left, swollen endosomes and lysosomes containing cellular debris in proximal tubules of control mice. Middle right, interstitial edema (asterisk) and a marginating polymorphonuclear leukocyte (arrow) adjacent to a severely injured proximal tubule in Ret^UB^ ^del^ kidney. Bottom, electron-dense cytoplasmic vacuoles, swollen endosomes, and lysosomes (arrowheads) in control and Ret^UB^ ^del^ mouse kidneys. Note the degenerating proximal tubular cell spilling cytoplasmic contents into the lumen in Ret^UB^ ^del^ mouse (arrow). (**C**) Ret^UB^ ^del^ mice have higher expression of kidney injury molecule 1 (Kim1) and more CD45^+^ leukocyte infiltration. The entire kidneys were scanned in tiles and stitched together (200× magnification, inset 400×). (**D**) Quantification of Kim1 expression and CD45^+^ cell infiltration by fluorescence integrated density using FIJI software. There is a significant increase in Kim1 expression (*P = 0.018, *n* = 5 per group) and CD45^+^ cell infiltration (*P = 0.019, *n* = 5 per group) in gentamicin-exposed Ret^UB^ ^del^ mice versus gentamicin-exposed controls using 1-way ANOVA followed by Tukey’s test for multiple comparisons. Gent, gentamicin.

Next, we tested whether adverse environmental exposures accelerated the development of CKD in mice with congenitally reduced nephron number. Premature infants are at increased risk of AKI in the neonatal period, with an incidence of 18%–48% in infants born at fewer than 36 weeks, and with higher rates of AKI associated with earlier gestational age (4). AKI is often multifactorial, and risk factors include exposure to nephrotoxins. Gentamicin is one of the most ubiquitous exposures, as preterm infants are at high risk of infection with bacteria susceptible to aminoglycosides (51, 52). Gentamicin enters proximal tubular cells by binding to the brush border (53, 54), followed by receptor-mediated endocytosis (55, 56). Gentamicin accumulates in lysosomes, causing swelling and rupture (57), releasing gentamicin and lysosomal enzymes into the cytosol, and causing further cell injury. However, it is not clear whether kidneys with low nephron endowment are more susceptible to gentamicin-induced injury. We injected neonatal Ret^UB^ ^del^ and littermate controls with gentamicin (100 mg/kg) or saline (4 μL/g) s.c. once a day for 7 days from P3 to P9. Kidneys were harvested 1 day after completing 7 days of treatment (P10). Of note, gentamicin total dose was lower in mice with low nephron number, given the weight-based dosing, as mice with low nephron number are slightly smaller than their littermate controls (Supplemental Figure 5). Gentamicin injection led to proximal tubular injury in both control and Ret^UB^ ^del^ mice. PAS-stained sections revealed proximal tubular vacuolization, which was more severe in Ret^UB^ ^del^ mice compared with littermate controls (Figure 5A). Immunostaining of Lamp1 showed that, under basal conditions, there is a similar pattern of Lamp1 expression in late endosomes and lysosomes under the brush border of proximal tubules in saline-injected controls and Ret^UB^ ^del^ mice, albeit with more endosomes and lysosomes in mice with low nephron number, as described in Figure 3D. However, after gentamicin exposure, Ret^UB^ ^del^ mice have more severely enlarged membrane vesicles in the proximal tubules, suggesting more endosome and lysosome swelling and engorgement after gentamicin exposure in mice with low nephron number (Figure 5A). To determine whether there was a difference in lysosome size between control and Ret^UB^ ^del^ mice after gentamicin exposure, we performed blinded human observer–based comparisons of Lamp1-expressing vesicle size. Given that lysosomal size varies among S1–S3 segments of the proximal tubules (58), we focused on the renal cortex where S1 and S2 segments were localized. We obtained 50 randomized images in each experimental group and performed 100 comparisons between groups. Our observer identified gentamicin-exposed Ret^UB^ ^del^ kidneys as having larger lysosomes than gentamicin-exposed controls in 85% of comparisons, which corresponded to a χ^2^ value of 24.5 and *P* < 0.001.

Further examination with electron microscopy revealed that, after gentamicin exposure, Ret^UB^ ^del^ and littermate controls had proximal tubular injury with vacuolated cytoplasm filled with endosomes, phagosomes, and lysosomes (Figure 5B). Early cell death was apparent, and areas of interstitial edema and inflammatory cell infiltrates were identified. Mitochondria were morphologically similar between normal and low nephron number groups (data not shown); however, there was mitochondrial rarefication in both groups, which could be secondary to damage and rupture or could be due to marginalization in the setting of profound lysosomal swelling and engorgement. While glomeruli were mostly intact, there were short segments of foot process effacement (Supplemental Figure 6).

The more severe vacuolization corresponded to higher expression of kidney injury molecule 1 (Kim1) and increased inflammation with CD45^+^ cell infiltration in Ret^UB^ ^del^ mice (Figure 5C). While quantification of Kim1 and CD45 infiltrates showed no difference between saline-injected control and Ret^UB^ ^del^ mice, gentamicin injection resulted in a significant increase in Kim1 expression in Ret^UB^ ^del^ mice compared with controls, with a mean Kim1 integrated density of 511 × 10^3^ in Ret^UB^ ^del^ compared with 76.25 × 10^3^ in controls (*P* = 0.018, 1-way ANOVA with Tukey’s test for multiple comparisons). Although CD45^+^ cells were increased in both groups, Ret^UB^ ^del^ kidneys had significantly more infiltrates than control kidneys, with a mean CD45 integrated density of 1,039 × 10^3^ in Ret^UB^ ^del^ mice compared with 264 × 10^3^ in controls (*P* = 0.019, 1-way ANOVA with Tukey’s test for multiple comparisons) (Figure 5D). These results indicate that low nephron endowment increases susceptibility to gentamicin-induced injury in the proximal tubules, likely due to increased proximal tubular endocytosis and uptake of gentamicin, given the robust-appearance of endosomes and lysosomes in Ret^UB^ ^del^ mice (Figure 3D and Figure 5A).

**Figure 6:**
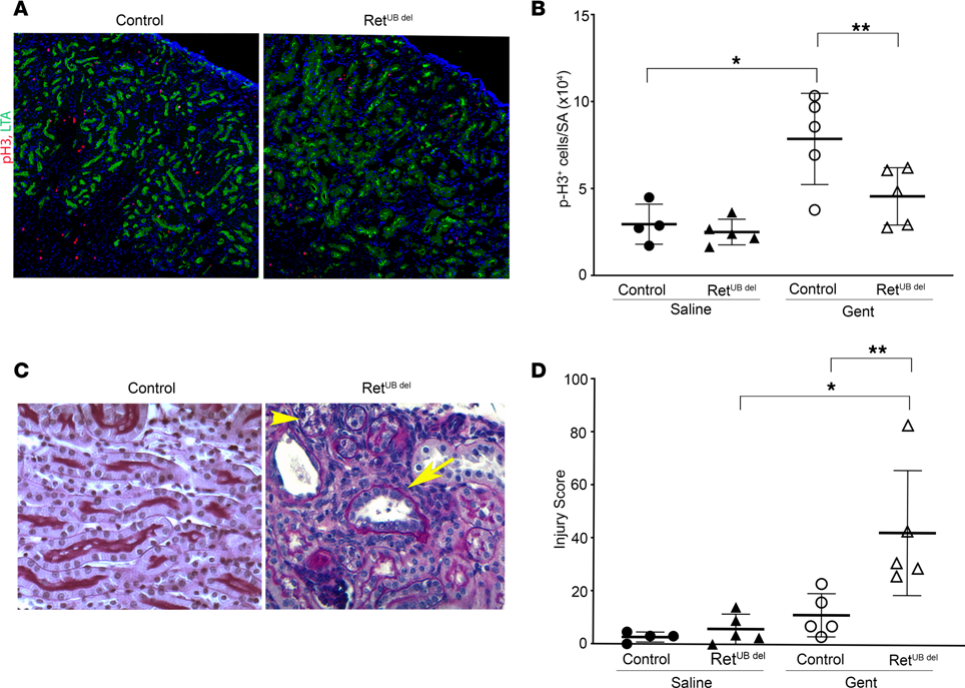
Neonatal Ret^UB^ ^del^ mice have incomplete renal repair after gentamicin-induced AKI. Newborn mice (control and Ret^UB^ ^del^) were injected with saline or gentamicin once a day for 7 days from P3 to P9. Kidneys were analyzed 1 day (P10) or 1 month after completing treatment. (**A**) Expression of phospho-histone H3 (pH3, red) in the proximal tubules labeled with LTA (green) in mice 1 day after completion of injections (200× magnification) (**B**) While saline-injected control and Ret^UB^ ^del^ mice show no difference in the baseline expression of pH3^+^ proliferating cells in the renal cortex and outer medulla, gentamicin injury induces a significant increase in the expression of pH3 in control mice (1-way ANOVA followed by Tukey’s test for multiple comparisons, *P = 0.015, *n* = 4–5 per group) but not in Ret^UB^ ^del^ kidneys (*P* = 0.28, *n* = 5 per group). Comparison of gentamicin-injected control and Ret^UB^ ^del^ kidneys also shows a significantly lower number of pH3^+^ cells in the cortex and outer medulla of Ret^UB^ ^del^ mice compared with controls (**P = 0.0377, *n* = 5 per group). (**C**) At 1 month following completion of gentamicin injections, control mice have near complete tubular repair, whereas Ret^UB^ ^del^ kidneys have areas of tubular atrophy (arrow), interstitial fibrosis, chronic inflammation, and residual tubular vacuolization (arrowhead). (**D**) Control mice had no significant increase in injury score 1 month after gentamicin injection (1-way ANOVA followed by Tukey’s test for multiple comparisons, *P* = 0.77, *n* = 4–5 per group), whereas Ret^UB^ ^del^ mice had significantly elevated injury scores following gentamicin-induced AKI (*P < 0.01, *n* = 5 per group, 1-way ANOVA followed by Tukey’s test for multiple comparisons). The difference between injury scores in control and Ret^UB^ ^del^ mice 1 month after gentamicin-induced AKI were also significant (**P < 0.01, *n* = 5 per group, 1-way ANOVA followed by Tukey’s test for multiple comparisons). Gent, gentamicin.

Studies on renal ischemic injury indicate that focal epithelial cell death and injury triggers tubular repair by proliferation of surviving cells (59, 60). We quantified proliferating cells by the expression of phospho-histone 3 (pH3) in the cortex and the outer strip of the outer medulla (S1–S3 segments of proximal tubule). While quantification of the number of pH3^+^ proliferating cells showed no difference in saline-injected control and Ret^UB^ ^del^ kidneys, gentamicin injection led to a significant increase in pH3^+^ cells in control kidneys, but not in Ret^UB^ ^del^ kidneys (Figure 6, A and B). This may be due to more severe epithelial injury resulting in fewer competent cells able to enter a proliferative state in the Ret^UB^ ^del^ kidneys. The low proliferative repair is associated with a more accelerated CKD phenotype, higher injury scores, and persistent inflammation 4 weeks after gentamicin injection (Figure 6, C and D). Our results indicate more severe long-term renal adverse effects following neonatal AKI in the setting of low nephron endowment. Given the persistence of inflammatory infiltrates in Ret^UB^ ^del^ mice, we reasoned that there may be an initial exaggerated inflammatory response to gentamicin in these mice.

### Ret^UB^ ^del^ mice have a unique inflammatory response to gentamicin-induced AKI.

**Figure 7:**
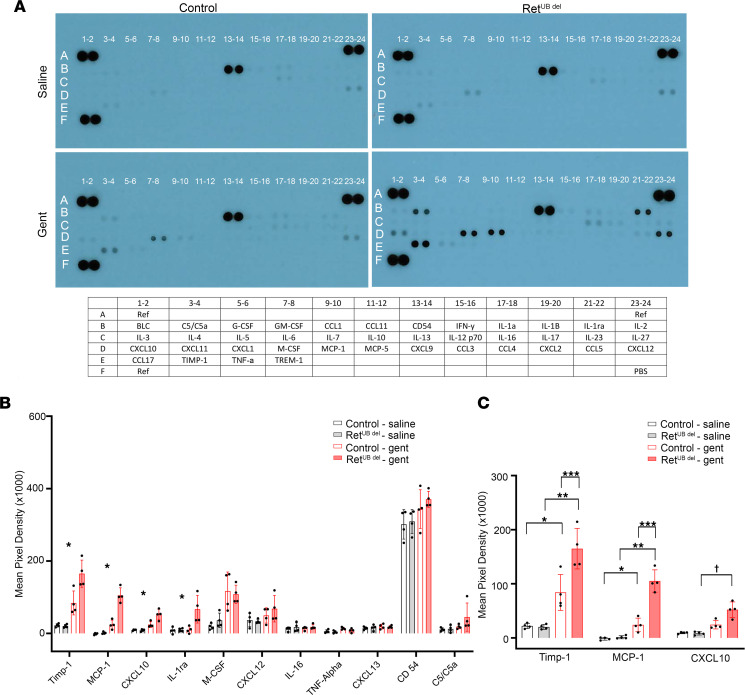
Ret^UB^ ^del^ mice exhibit a unique inflammatory response to gentamicin-induced AKI. Newborn mice (control and Ret^UB^ ^del^) were injected with saline or gentamicin at 100 mg/kg once a day for 7 days at P3–P9. Kidneys were analyzed 1 day after the completion of injections (P10). (**A**) Representative images of cytokine arrays of kidney homogenates. Cytokines are identified in the chart below the array membrane. (**B**) Of 40 total cytokines tested, 11 cytokines that had detectable expression following saline or gentamicin injection are shown, and levels of Timp-1, MCP-1, CXCL10, and IL-1ra are significantly different between groups tested by 1-way ANOVA (*P < 0.01, *n* = 4 per group). (**C**) One-way ANOVA followed by Tukey’s test for multiple comparisons indicates significant differences in levels of Timp-1 and MCP-1 in saline versus gentamicin injected controls as well as in saline and gentamicin exposed Ret^UB^ ^del^ mice (*P < 0.05, **P < 0.0001, *n* = 4 per group). Note higher expression of Timp-1 and MCP-1 in gentamicin-injected Ret^UB^ ^del^ compared with gentamicin-injected control mice (*** *P* ≤ 0.003). CXCL10 was uniquely elevated in Ret^UB^ ^del^ mice after gentamicin injection (^†^*P* < 0.0001). Gent, gentamicin.

**Figure 8:**
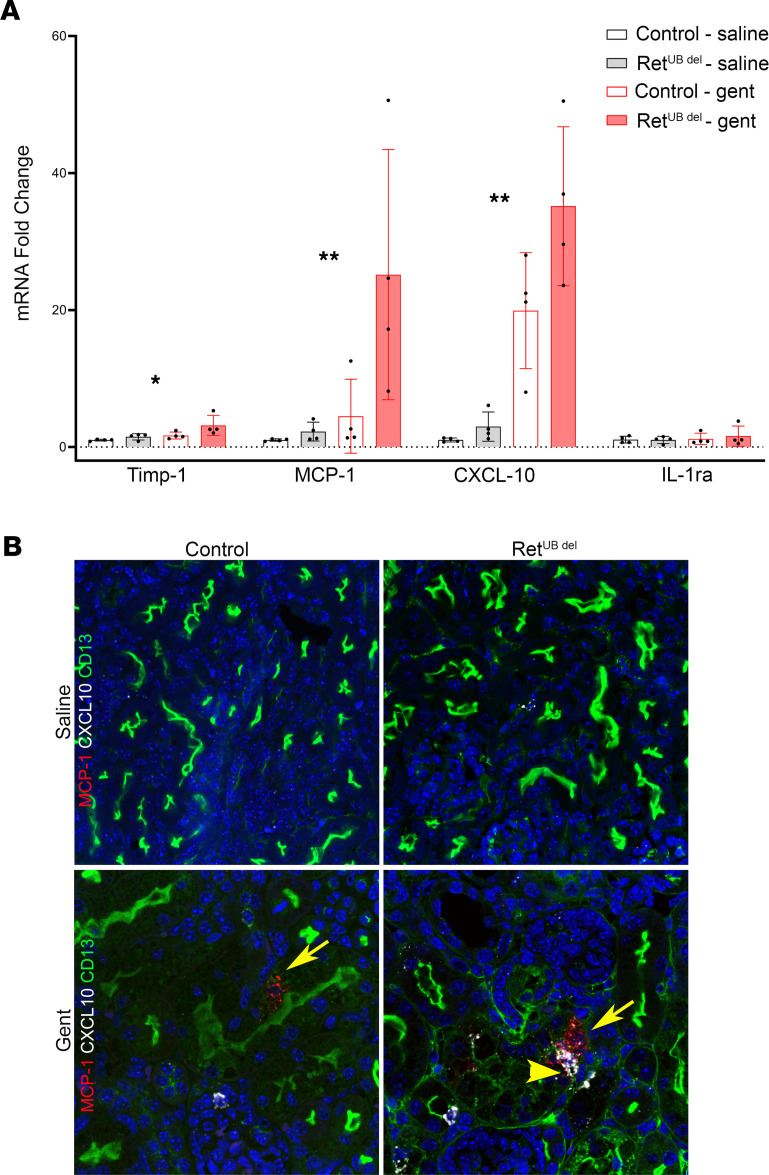
Expression of CXCL10 and MCP-1 in gentamicin-injured proximal tubular cells. (**A**) qPCR analysis shows significantly different mRNA expression levels for Timp-1, MCP-1, and CXCL10 (1-way ANOVA, *P < 0.05), with higher MCP-1 and CXCL10 mRNA levels in Ret^UB^ ^del^ mice compared with controls after gentamicin injection (1-way ANOVA followed by Tukey’s test for multiple comparisons, **P < 0.05, *n* = 4 per group). (**B**) RNAscope using probes to CXCL10 (white) and MCP-1 (red) was coupled with immunostaining of CD13 (green) to identify proximal tubules in kidneys 1 day after completing saline or gentamicin injections. In the saline-exposed groups, there was minimal expression of CXCL10 and MCP-1 (top panel). Gentamicin injection resulted in the increase of mRNA of MCP-1 (arrow) in the damaged proximal tubules of both control and Ret^UB^ ^del^ kidneys and in the increased expression of CXCL10 in Ret^UB^ ^del^ tubules (arrowhead) (630× magnification). Gent, gentamicin.

The importance of intact proximal tubular structure and function on the integrity of interstitial compartment has been well demonstrated (61, 62). Tubular epithelial damage and the resulting molecular response can trigger interstitial inflammation. Cytokines are major players in the complex interplay between damaged tubules and inflammatory and immune cells (63). To identify inflammatory mediators in the injured kidney, we used a commercially available Proteome Profiler Mouse Cytokine Array Kit (R&D, ARY006) for simultaneous detection of 40 cytokines, chemokines, and acute phase reactants in mouse kidney homogenates 1 day following the completion of gentamicin or saline injection. Under basal conditions (saline-exposed, age-matched mice), there were no differences in cytokine expression between Ret^UB^ ^del^ mice and controls, suggesting that there is no inflammatory response associated with low nephron number in mice at P10. In contrast, we detected the expression of 11 cytokines in the kidneys of control and Ret^UB^ ^del^ mice following gentamicin injection. Among them, Timp-1, MCP-1, CXCL10, and IL-1ra were significantly higher in gentamicin-exposed Ret^UB^ ^del^ kidneys compared with gentamicin-exposed littermates with normal nephron number (Figure 7). Given that Timp-1, MCP-1, and CXCL10 have all been implicated in renal inflammatory diseases (64–79), we focused on these cytokines for further analysis. We found that, while both control and Ret^UB^ ^del^ mice had significantly increased expression of Timp-1 and MCP-1 after gentamicin, the increase was much greater in Ret^UB^ ^del^ mice. Interestingly, CXCL10 was uniquely elevated in Ret^UB^ ^del^ mice after gentamicin (Figure 7C). Quantitative PCR (qPCR) analysis from kidney homogenates showed increased expression of mRNA for Timp-1, MCP-1, and CXCL10 in gentamicin-exposed Ret^UB^ ^del^ mice, confirming their expression in cells of renal origin and/or intrarenal infiltration (Figure 8A).

To determine the location of cytokine producing cells, we performed RNAscope using probes to Timp-1, MCP-1, and CXCL10, and we colabeled proximal tubules using antibody to CD13 (ACD, RNA Protein Codetection Assay). We discovered that, while all 3 cytokines were produced in interstitial cells (data not shown), MCP-1 and CXCL10 were also produced by the damaged proximal tubular epithelial cells (Figure 8B). Although there was detectable MCP-1 and CXCL10 mRNA signal in tubules of both control and Ret^UB^ ^del^ mice, there was more signal detected in mice with low nephron number, which is consistent with the higher protein and mRNA levels shown by the cytokine array and qPCR analysis.

In summary, mice with congenitally reduced nephron number develop glomerular and proximal tubular hypertrophy by 6 weeks of age, which — while initially adaptive — becomes maladaptive with the emergence of a CKD phenotype by 6–12 weeks of age. Increased cellular stress secondary to nephron hypertrophy may accelerate the development of CKD in mice with low nephron endowment. As neonates, Ret^UB^ ^del^ mice are more susceptible to gentamicin-induced AKI, with more severe injury, a profound and unique inflammatory response, and incomplete repair with rapid progression to CKD. Our studies highlight the vulnerability of the kidney with low nephron endowment, and mice generated in this study are useful for the study of pathogenesis of kidney disease in humans born preterm.

## Discussion

We generated mouse models of 30%–70% nephron reduction that simulate human premature kidneys by manipulating Ret tyrosine kinase activity or expression during late gestation. While early delivery would be an ideal experimental design to study prematurity related human kidney disease, delivering mice more than 2 days before natural birth does not yield viable animals (80). Mice delivered shortly prior to natural delivery results in mice with a 20% nephron deficit (28); however, this does not recapitulate the spectrum of nephron number seen in humans born preterm — particularly, the significant nephron reduction seen in extremely preterm infants (10). Although Ret signaling is not known to play a role in renal growth and maturation beyond the period of nephrogenesis, interfering with Ret signaling could result in subtle phenotypic changes not recognized in this study. While deleting *Ret* in our genetic model is permanent, in our chemical model, Ret tyrosine kinase activity is only transiently inhibited. Therefore, if Ret were necessary for further renal growth and development, kidneys in the chemical model would not be affected. In addition, UB tips that have *Ret* deletion are often replaced by overgrown WT cells (81), so in the case of *Ret* deletion, any potential role of Ret in renal growth and maturation after nephrogenesis is unlikely to be affected by deletion of *Ret* in a fraction of fetal UB cells. Future analysis including detailed cell type and gene expression profiling may reveal subtle phenotypic changes. It is also important to note that, while humans with hypomorphic *Ret* mutations may have low nephron endowment, the mutations can cause other developmental defects such as Hirschsprung’s disease, renal dysplasia, and urinary tract anomalies (82, 83) because Ret signaling is affected from the beginning of organogenesis. In contrast, perturbation of Ret signaling late in gestation causes no significant disturbance of the enteric nervous system, and there is no evidence of renal dysplasia or hydronephrosis, likely because treatment begins after the critical period of Ret-dependent development of urinary tract and enteric nervous system.

Of the 2 models generated, Ret^UB^ ^del^ mice generated by UB-specific deletion of *Ret* are more consistent in the range of nephron reduction. Interestingly, we found that the loss of Six2^+^ progenitor niches was patchy, suggesting that *Ret* deletion following Dox administration may not be uniform. It is possible that cells in which *Ret* is successfully deleted do not form branching tips (81), leaving areas with no surrounding Six2^+^ cells; areas in which *Ret* is not deleted continue to branch, and Six2^+^ niches form, resulting in a patchy appearance of Six2^+^ progenitor niches.

Our study directly links low nephron number with compensatory glomerular and proximal tubular hypertrophy. While several human studies have shown an inverse correlation between nephron number and glomerular size (84, 85), no studies have demonstrated that this is an acquired process, nor have any studies outlined a timeline for the development of compensatory hypertrophy. We show that mice with low nephron number have normal glomerular size at 2 weeks of age but that they embark on an accelerated growth trajectory between 2 and 6 weeks of age. Human autopsy studies corroborate the clinical relevance of this model. In studies of humans born preterm who developed chronic kidney disease later in life, glomeruli appeared enlarged (86). In addition, we have shown that hypertrophy extends beyond glomeruli to tubules. We found that proximal tubules are hypertrophied with a direct correlation between glomerular surface area and mean tubular diameter. This is in agreement with the early report by Oliver et al. showing a close relationship between the size of the glomerulus and the proximal tubules in kidneys where small glomeruli are connected to small tubules, normal glomeruli to normal tubules, and larger glomeruli to larger tubules (48), suggesting that nephron hypertrophy is linked to absorptive workload that is in parallel with glomerular filtration.

Compensatory hypertrophy in kidneys with low nephron number is thought to be due to increased single-nephron GFR. Brenner et al. (13) first hypothesized that, in the setting of increased single-nephron GFR, there is an adaptive compensatory hypertrophy that becomes maladaptive. In this study, we show that maladaptive changes emerge by adolescence in mice with congenitally reduced nephron number. By 12 weeks, mice develop evidence of decreased renal function, as shown by increased sCr and urine albumin excretion. By 6–12 weeks, they have tubular atrophy, interstitial fibrosis, and inflammatory cell infiltrates, suggestive of the development of CKD. By 9 months of age, there are glomerular changes of evolving FSGS. These features are reminiscent of clinical findings in young adults with a history of preterm birth and low birthweight who were found to have proteinuria and elevated creatinine and who underwent renal biopsies. These individuals were found to have oligomeganephronia, glomerulosclerosis, mild tubular atrophy, and patchy fibrosis (86).

In contrast to congenitally low nephron number, uninephrectomy in adult mice did not result in the same degree of renal hypertrophy, and there were no changes to suggest CKD during the 4-week study period. This is in agreement with other reports showing less compensatory growth when nephrectomy occurred in adult rodents (87). One previous study demonstrated that neonatal nephron loss in rats (removal of 1 kidney day 1 after birth, when nephrogenesis is ongoing) resulted in compensatory renal growth 4 weeks later with larger glomerular perimeters and more cells per glomerular cross section (88). Conventional wisdom suggests that the progressive increase in metabolic demand during somatic growth drives this postnatal growth. Indeed, human autopsy studies in White American males showed that marked glomerular hypertrophy in kidneys with low nephron number was closely associated with high body surface area (89), supporting metabolic demand as a driving force for renal compensatory growth. However, in neonatal nephrectomized rats (88) and in our model of congenitally low nephron number, early adaptive growth was associated with the development of renal pathologic changes of CKD. At this time, the cellular and molecular mechanisms of glomerular and tubular hypertrophy, as well as the cellular basis for the transition from compensatory hypertrophy to maladaptive changes resembling CKD, are not fully understood. Future studies with single-nucleus RNA-Seq in conjunction with spatial transcriptomics may reveal transcriptional programs regulating renal hypertrophy and the development of CKD.

We used Ret^UB^ ^del^ mice to develop a model of preterm neonatal AKI using gentamicin as a nephrotoxic exposure in the first few days of life. Mice with congenitally decreased nephron number experience more severe AKI after gentamicin and have a higher level of the proinflammatory cytokines/chemokines MCP-1, CXCL10, and Timp-1. While MCP-1 and Timp-1 were elevated in controls exposed to gentamicin, CXCL10 was only elevated in mice with low nephron number exposed to gentamicin. In addition, MCP-1 and Timp-1 levels were much higher in Ret^UB^ ^del^ mice treated with gentamicin compared with controls under the same treatment conditions. To determine the origin of MCP-1, CXCL10, and Timp-1 after injury, RNAscope was performed in conjunction with immunostaining with antibody to CD13 to identify proximal tubular cells. We found that, while all 3 cytokines were produced in interstitial cells, MCP-1 and CXCL10 were also produced in the damaged proximal tubules. MCP-1 and CXCL10 were detected in both control and Ret^UB^ ^del^ mice exposed to gentamicin; however, there were higher mRNA levels in mice with low nephron number as confirmed by qPCR analysis.

MCP-1 is a monocyte chemotactic factor that binds CCR2 on monocytes and promotes monocyte mobilization from bone marrow, recruitment to local tissues, and differentiation into macrophages (73, 90). MCP-1 has been implicated in inflammation and kidney diseases (73, 75, 79, 91). CXCL10 is also increased after injury in Ret^UB^ ^del^ kidneys. CXCL10 is a chemokine induced by IFN-γ. It binds the chemokine receptor CXCR3 on CD4^+^ and CD8^+^ lymphocytes, recruits T cells to inflammatory sites, and promotes effector activity. It also is a chemoattractant for macrophages, monocytes, and NK cells (92, 93). CXCL10 has been implicated in renal transplant rejection, and increased urinary CXCL10 is associated with tubulointerstitial inflammation and transplant rejection (64, 77, 94, 95). Timp-1 is a metalloproteinase inhibitor that also has many functions, including cell proliferation and growth, apoptosis, angiogenesis, and inhibition of smooth muscle cell migration (96). Timp-1 has also been explored as a biomarker of human AKI (68). Elevated levels of MCP-1, CXCL10, and Timp-1 in Ret^UB^ ^del^ mice after AKI suggests a more severe inflammatory response in mice with low nephron number, yet the functional impact of this dysregulated cytokine response remains untested in this study. Understanding cytokine function in this context will be an important next step to validate the role of inflammation after AKI in mice with low nephron number. The presence of such cytokines in human studies and the fact that they are well-known chemoattractants raises the possibility of therapies aimed at blocking the inflammatory loop in human preterm AKI.

The reason for increased inflammation and more severe injury with poor repair after gentamicin in mice with low nephron number is not fully understood. Our basic understanding of tubular repair is largely obtained from animal models of acute ischemia reperfusion injury (IRI) when kidneys are deprived of blood flow, typically for 15–45 minutes, and then reperfused. This type of insult causes the most severe injury to the S3 segments localized to the outer stripe of outer medulla, although other segments of the nephron and collecting ducts sustain injury as well. Cell injury, death, and detachment from the tubular basement membrane typically occur in a focal and mosaic fashion even within the same tubular segment. Genetic labeling and lineage-tracing studies indicate that a subset of surviving tubular cells responded to injury with transcriptional activation and reenter the proliferative state to cover the denuded area and redifferentiate to establish intercellular junctions with neighboring cells (59, 60, 97, 98). In contrast, the source of cells for tubular repair after gentamicin-induced injury is less studied. More than 50 years ago, electron microscopic examinations in rats injected with gentamicin (40 mg/kg, daily for 14 days) suggested that regenerating cells appeared to originate from residual epithelial cells (99). Our current study indicates that gentamicin causes more generalized and profound tubular injury along the entire length of proximal tubules in mice with low nephron endowment. It is conceivable that there are fewer cells with the capacity to activate reparative program and reenter the cell cycle for tubular repair. The reduced repair, prolonged injury, and more severe interstitial inflammation can all account for the accelerated CKD phenotype in underdeveloped kidneys injured by gentamicin.

It is possible that reduced renal mass results in higher exposure to gentamicin per cell, causing increased toxicity in mice with low nephron number. We attempted to achieve a similar volume of distribution of gentamicin in mice with normal and low nephron number by giving the drug dose based on body weight. Whether toxicity is due to innate characteristics or responses to injury in kidneys with low nephron mass needs to be further explored. In this study, we chose the dose of gentamicin by extrapolating from studies of adult rodents with gentamicin-induced AKI (100, 101) and this dose is higher than the dose given to preterm infants per kilogram of body weight. Therefore, caution must be taken when interpreting these results. In the clinical setting, while gentamicin doses are lower, infants also experience concurrent renal insults such as infections, hemodynamic instability, and/or other nephrotoxic exposures, which can all contribute to renal injury or worsen the nephrotoxic effects of a lower dose of gentamicin. Further understanding of cellular and molecular mechanisms of injury and cell death — and, more importantly, tubular stress resistance and repair — following gentamicin use is essential in order to develop measures to reduce its renal toxicity in premature infants.

This study highlights the vulnerability of the preterm kidney. In our model of low nephron endowment, the risk of maladaptive changes and the development of CKD — in the absence of episodes of acute injury — is high. In addition, AKI from a commonly prescribed medication, gentamicin, further exacerbates renal injury with incomplete repair and accelerates the development of CKD. Given that up to 50% of extremely preterm infants will experience AKI in the neonatal period and a vast majority will be treated with gentamicin, this population is at extremely high risk for future renal dysfunction. A recent publication by the AKI!NOW Steering Committee (102) highlights the importance of clinical care of patients who have recovered from critical illness and AKI. It emphasizes the need for ongoing research to improve outcomes in vulnerable populations, including infants discharged from the neonatal intensive care unit, as these children will grow into adulthood facing increased risk of CKD.

## Methods

Supplemental Methods are available online with this article.

### Animal studies.

*Ret^flox-V805A^* (The Jackson Laboratory, 028548) female and male mice were bred for timed pregnancy. To inhibit Ret tyrosine kinase activity, pregnant female mice were injected i.p. with NA-PP1 (Medchem express, HY-13941/CS-1804) or vehicle (cremaphor/saline/ethanol in 1:7:2 ratio) from E16.5 through E18.5 at the dose of 32.25 mg/kg, 50 mg/kg, and 62.5 mg/kg, respectively. Pups delivered spontaneously E19.5. NA-PP1 was prepared using published methods (37). Both male and female offspring were used for experiments. To delete *Ret* in the UB, *Tet-O-Cre* (103) and *Hoxb7rtTA* (The Jackson Laboratory, 036718) mice were crossed into *Ret^flox-V805A^* mice. *Tet-O-Cre;Hoxb7rtTA;Ret^flox-V805A^* mice were bred for timed pregnancy. Pregnant females were given Dox (Henry Schein, 1315046; 2 mg/mL) dissolved in drinking water beginning E15.5, E16.5, or E17.5 through delivery. Several breeders were heterozygous for *Hoxb7rtTA* and yielded littermate controls without *Ret* deletion (*Tet-O-Cre; Ret^flox-V805A^*). Offspring were genotyped through Transnetyx core service. Pups with the genotype of *Tet-O-Cre;Hoxb7rtTA;Ret Ret^flox-V805A^* who were exposed to Dox in utero with resultant *Ret* deletion in the UB were named Ret^UB^ ^del^.

### Neonatal AKI with gentamicin.

AKI was induced by injecting gentamicin (100 mg/kg, Henry Schein, 54894) or saline (4 μL/g) s.c. P3-P9. Kidneys were harvested 1 day or 1 month after completing injections.

### Quantification of glomerular number.

Entire kidneys were harvested, decapsulated, cut into 2 mm^3^ pieces, and incubated in 5 mL of 6N Hydrochloric acid at 37°C for 35 minutes with gentle shaking, followed by pipetting to dissociate glomeruli from the surrounding tissue. Digestion was terminated with 25 mL of sterile water and a number of glomeruli in 1 mL of digested kidney was counted in duplicate (or triplicate, if duplicates differed by > 10%) (42).

### Quantification of glomerular and tubular size.

Kidneys were harvested at 2, 6, or 12 weeks. Formalin-fixed, paraffin-embedded kidneys were sectioned at 4 μm thickness. PAS-stained sections were scanned at 200× magnification using Aperio ImageScope from Leica Biosystems, and image analysis was performed using QuPath software (v0.2.2). Glomerular surface area was measured manually by a blinded investigator by outlining the entire glomerular basement membrane area of the glomerular globe, excluding Bowman’s capsule, and using QuPath software to measure surface area in the measured plane. All glomeruli in the scanned kidney section were measured (range 50–200 glomeruli per kidney) to reduce sampling bias. The narrowest proximal tubular profiles in the outer strip of the outer medulla were selected, and diameter was manually measured. In total, 50 tubules in half kidney sections and 100 tubules in entire kidney sections were measured. All measurements were performed by an investigator blinded to mouse genotypes and treatment. Tubular size and glomerular surface area were analyzed using mixed-effects regression models accounting for within-subject correlations of tubular diameter or log glomerular surface area. All mean values reported for glomerular surface are geometric means.

### Electron microscopy examination.

Transmission EM studies were performed using the standard method, and images were acquired with a JEOL JEM-1011 electron microscopy equipped with a Gatan digital camera.

### RNAscope.

Kidneys were fixed in formalin for 24 hours followed by paraffin embedding and sectioning at 8 μm thickness. RNAscope was performed according to manufacturer’s instructions (ACD, RNAscope with IHC codetection kit), using a 15-minute target retrieval and protease plus for protease digestion. Fluorophores were diluted 1:1,500 (Akoya Biosciences). Sections were colabeled using anti-CD13 antibody (1:800). Samples were imaged on a Zeiss Axio Observer CSU-X spinning disc confocal microscope with a 63×/1.4 NA objective.

### Statistics.

The number of animals is indicated for each experiment. All ELISA and urinary creatinine tests were performed in duplicate. All qPCR was performed in triplicate. Data are presented as the mean with 95% CI or SD. Welch’s 2-tailed *t* test was used to determine the statistical significance between 2 groups. Comparisons of multiple means were performed with 1-way ANOVA followed by Tukey’s test for multiple comparisons. The size of Lamp1 positive objects was analyzed using blinded, observer-based comparisons between groups. Data was analyzed with χ^2^ test. For all studies, a *P* value of less than 0.05 was considered significant.

To determine the mean glomerular surface area and mean tubular diameter, we used mixed effects regression models to account for the multiple measurements within each mouse. Glomerular surface area has an approximate log-normal distribution; thus, the dependent variable in models involving glomerular surface area is its natural logarithm. Mean values of glomerular surface that we present are predicted geometric means generated by exponentiation of model results. We used similar mixed-effects models to generate predicted arithmetic means for tubular diameter. Statistical analysis was performed with Prism software (version 8.1.2) and R (R Core Team 2021; R Foundation for Statistical Computing, Vienna, Austria; https://www.R-project.org/). A *P* value of less than 0.05 was considered significant.

### Study approval.

All procedures involving mice were conducted according to the NIH’s *Guide for the Care and Use of Laboratory Animals* (National Academies Press, 2011) and approved by the IACUC of Columbia University.

## Author contributions

PIG designed and performed the majority of experiments, analyzed data, and wrote the manuscript. LL, HAH, ISH, and KX helped with the experiments and acquired and analyzed data. DAB performed the majority of statistical analyses. MR advised on studies using NA-PP1 and the analysis of enteric nervous system. FC and QAA advised on study design and data interpretation and edited the manuscript. VDD provided expertise and insightful discussions of mouse kidney histopathology and edited the manuscript. FL designed and directed the study, performed experiments, and edited the manuscript.

## Supplementary Material

Supplemental data

## Version 1 01/10/2023

In-Press Preview

## Version 2 02/08/2023

Electronic publication
