# Supplementary material for: Low nephron endowment increases susceptibility to renal stress and chronic kidney disease
Source: JCI Insight. 2023 Feb 8;8(3):e161316. doi: 10.1172/jci.insight.161316 (PMC9977438; doi:10.1172/jci.insight.161316)
Supplement: Supplemental data [file jciinsight-8-161316-s136.pdf]

Supplemental Methods:

Immunohistochemistry, RNA scope, and antibody information: For immunohistochemistry, kidney sections were fixed with 4% PFA in PBS for 4 hours for adults or 24 hours for mice $\leq$ 2 weeks of age, permeabilized with 0.1% Triton X-100 in PBS for 30 min, then blocked with 10% goat serum and 0.5% bovine serum albumin (BSA) in PBS. Incubations with primary antibodies were carried out at 4 °C overnight. After washing, the sections were further incubated for 1 h at room temperature with the appropriate secondary antibodies. Nuclei were counterstained with DAPI (Vector, H-1200). Sections were visualized with a Zeiss AxioObserver.Z1 inverted fluorescence microscope or Zeiss Axio Imager M2 photographed with a digital camera, and analyzed with Axiovision software or FIJI software.

The following primary antibodies were used:

Antibody	Vendor	Catalogue Number	Dilution
Calbindin	Millipore	PC253l	1:300
CD45	Life Tech	MCMD4500	1:200
CD13	Abcam	ab108310	1:800
Collagen I	Southern Biotech	1310-01	1:200
Endomucin	Santa Cruz	SC-65495	1:250
HuC/HuD	Abcam	ab184267	1:1500
Kim1	R&D Systems	AF1817	1:800
Laminin	Sigma	L9393	1:1000
Lamp1	Abcam	ab25245	1:400
PH3	Abcam	ab1791	1:1000
Ret	Abcam	Ab134100	1:1000
Six2	ProteinTech	11562-1-AP	1:300
NK-ATPase	Bioss	bsm-52485R	1:800
THP	Biotechne/R&D Systems	AF5175	1:400
TSC	Mark Knepper/NIH		1:200
NFH	Millipore	AB5539	1:2500

Secondary antibodies included Alexa Fluor 488-conjugated goat anti-rabbit, goat anti-rat or donkey anti-goat IgG, and Alexa Fluor 594-conjugated donkey anti-rabbit or anti-rat IgG and goat anti-chicken IgG (Molecular Probes, A-11070, A-11006, A11055. A11042, A21029, A-11042; 1:200-1:400).

Proximal tubules were stained using fluorescein-conjugated lectin Lotus tetragonolobus agglutinin (FITC-LTA, FL-1321, Vector, 1:800) or Biotin-conjugated LTA (BIOTIN-LTA, B1352, Vector, 1:800) then bound to streptavidin-conjugated to Cy5 (STREPTAVIDIN-649, SA-5649, Vector, 1:800). Collecting ducts were stained with fluorescein-conjugated lectin dolichos biflorus agglutinin (DBA-FITC; FL-1031, Vector 1:800).

Probes for RNAscope include Mm-Timp1-c3 (316841-C3), Mm-CCL2 (311791) and Mm-Cxcl10-C2 (408921-C2).

Fluorophores for RNAscope include OPAL 520, OPAL 690, and OPAL480 (FP1487001KT, FP1497001KT, FP1495001KT).

Quantification of Collagen I, CD45, Kim 1 expression: Kidney sections were immunostained with antibodies to CD45, Collagen I or Kim1 and entire kidney sections were traced at 5x magnification and imaged at 200x magnification under a Zeiss microscope (Axio Imager M2, Zeiss) equipped with a Hamamatsu camera (Orca-R2, Hamamatsu). Images were stitched together using NIS-Elements before analysis of integrated density with FIJI software.

Quantification of phospho-histone 3 (p-H3): Kidney sections were immunostained with antibodies to phospho-histone 3 and co-labeled with LTA conjugated to FITC and entire kidney sections were traced at 5x and imaged at 200x magnification as described above. The number of p-H3 positive cells in the cortex and outer strip of the outer medulla were counted and normalized to surface area using FIJI software.

Quantification of Lamp-1 expression: Immunohistochemistry of kidney sections was performed with antibodies to Lamp1 and NK-ATPase and co-labeled with LTA to identify proximal tubules. Fifty random images per kidney were taken at 63x in the cortical region. Proximal tubules were identified as regions of interest. Lamp1 integrated fluorescent density was measured using FIJI software, and corrected for the number of nuclei in identified regions of interest (proximal tubules).

Whole mount immunostaining and image analysis: P1 kidneys were harvested, fixed in 4% PFA at 4°C for 24 hours, and then transferred to PBS. Immunostaining was performed with primary antibody to calbindin or Six2 (3 day incubations) and secondary antibody incubations were 3 days. Kidneys were optically cleared using established methods (1), photographed with Nikon Ti Eclipse inverted microscope with A1 scanning confocal unit at Columbia University Medical Center Confocal and Specialized Microscopy Core.

Immunoblot analysis: Pregnant $Ret^{flox\text{-}V805A}$ females were injected with vehicle or NA-PP1 on E16.5 and E17.5. Pups were sacrificed and kidneys were harvested 4 hours after injection on E17.5. For protein extraction from kidneys, RIPA buffer (Thermo Scientific, 89900) was used in conjunction with protease inhibitor cocktail (Thermo Scentific, 78430) and phosphatase inhibitor cocktail (Thermo Scientific, 1862495). Immunoblots were probed with antibodies to Ret (Abcam, Ab134100, 1:1000) followed by incubation with the appropriate secondary antibody conjugated with horseradish peroxidase (HRP, Life Technologies, 1:5000). Actin (Sigma, SAB4301137, 1:1000) was used as a loading control.

Cytokine Array: Kidneys were harvested 1 day after completion of gentamicin treatment as outlined previously (P10) and cytokine array was performed using the Proteome Profiler Mouse Cytokine Array Kit (R&D, ARY006) according to manufacturer's specifications. Protein was isolated with PBS containing protease inhibitors Aprotinin (Tocris, 4139), Leupeptin (Tocris, 1167) and Pepstatin (Tocris, 1190) with Triton X-100 (Sigma T9284). A total of 300 µg of protein was loaded per array.

Dextran injection: Texas Red conjugated dextran (70kD, Invitrogen, D1830) was injected into the tail vein of adult mice 3 minutes prior to sacrifice, kidneys were co-labeled with antibody to endomucin as outlined above.

Real-time RT-PCR analysis. Total RNA was isolated from kidneys using the Molecular Pathology Shared Resource Core at CUIMC. DNA was eliminated from 1 µg of RNA with deoxyribonuclease I (DNase I, Invitrogen, 18068-015). Post-DNase I digestion, 1 µg of RNA was applied for reverse transcription with iScript™ cDNA Synthesis Kit (Bio-Rad, 1708890). Each time, 100 ng of cDNA and 500 nM primers were used for real-time PCR in a 10-µl reaction mixture with iQ SYBR Green Supermix (Bio-Rad, 170-8880) using

a Bio-Rad™ CFX96TM Real-Time PCR System. Primers were designed with Integrated DNA Technologies Primer Quest Tool. The sequence of each primer is as follows: MCP1, forward: 5′-AGGTCCCTGTCATGCTTCTG-3′ and reverse: 5′-TCTGGACCCATTCCTTCTTG-3′; Timp1, forward: 5′-AGGTGGTCTCGTTGATTT -3′ and reverse: 5′-GTAAGGCCTGTAGCTGTGCC-3′; IL-1ra, forward: 5'-ATGGAAATCTGCTGGGGACC-3' and reverse: 5'-CTATTGGTCTTCCTGGGAGT-3'; CXCL10, forward: 5'-CCCACGTGTTGAGATCATTG-3' and reverse: 5'-CAGTTAAGGAGCCCTTTTAGACC-3'; *18s* rRNA forward: 5′-GTAACCCGTTGAACCCCATT-3′ and reverse: 5′-CCATCCAATCGGTAGTAGCG-3′. *18s* rRNA was used as an internal control. The expression of each specific gene relative to *18s*rRNA was calculated using the Pfaffl method (2).

***Analysis of enteric nervous system**:* Distal colons were harvested and 1 cm^2 sections were removed, cut along mesenteric boarder, and pinned flat for fixation in 4% PFA for 2 hours at 4° C prior to transferring to PBS. Immunostaining was performed by incubating with antibody to HuC/HuD or NFH for 72 hours and then secondary antibody for 24 hours before image analysis as previously published (3).

***Urine and blood analyses**.* Urinary albumin excretion was measured with the mouse albumin ELISA kit (Crystal Chem, 80630) and urine creatinine was measure with the mouse creatinine kit (Crystal Chem, 80350) following the manufacturer's instructions. Serum creatinine was measured at Yale O'Brien Kidney Center using a targeted Liquid Chromatography-Multiple Reaction Monitoring (LC-MRM) workflow on a 4000 QTRAP mass spectrometer. Mass spectral data are collected and analyzed/quantified using Analyst software (v.1.5).

***Tubular injury score and quantification of tubular injury and renal inflammation**:* Ten random images from the cortex to the outer stripe of the outer medulla for each kidney were PAS-stained and scanned at 100x magnification using Aperio ImageScope from Leica Biosystems and FIJI software (NIH) was used to obtain tubular injury scores. A graticule grid was randomly placed on each picture to generate 96 grids. Histology of tubular profiles of each grid was carefully examined, and a score 0 or 1 was given for each grid: 0=normal histology; 1=tubular vacuolization, tubular atrophy, casts in the tubular lumen, and interstitial inflammatory cells infiltration. The injury score was calculated by adding all 96 grids from each image and all

10 images from each kidney. All measurements were performed by an investigator blinded to mouse genotypes and treatment.

Supplemental Figures:

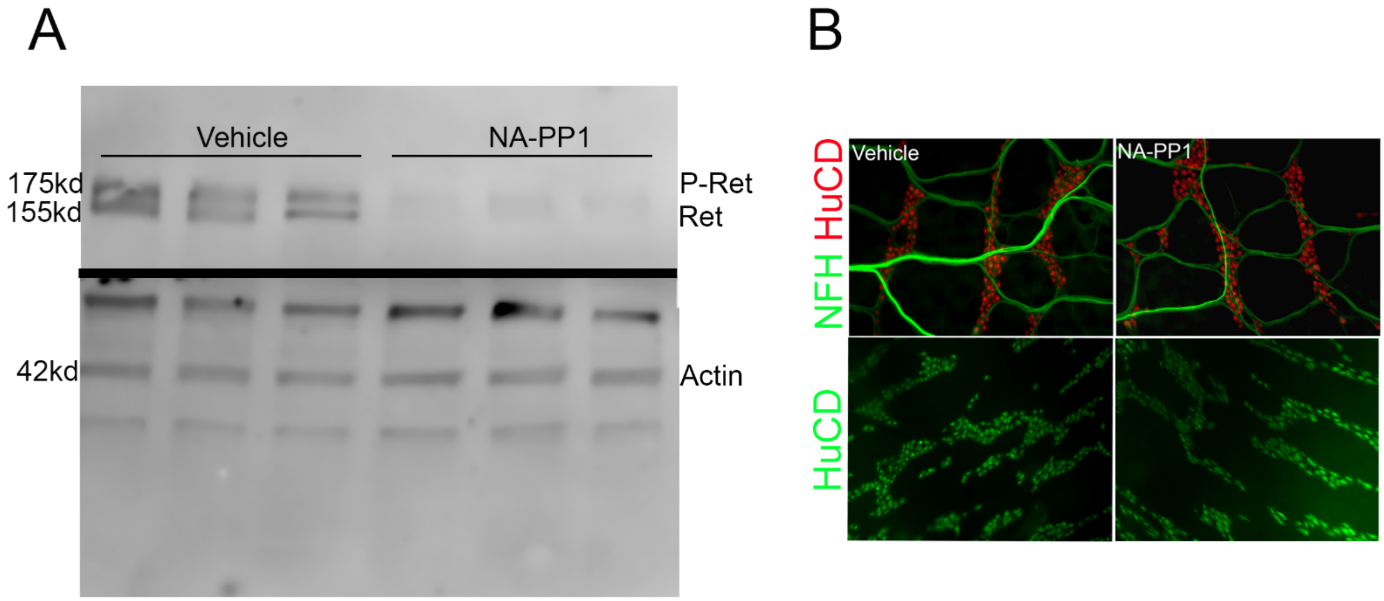

Supplemental Figure 1: A. Western blot of protein homogenates from vehicle exposed and NA-PP1 exposed kidneys harvested on E17.5. Pregnant Ret$^{flox\text{-}V805A}$ mice were injected daily with vehicle or a small molecule Ret tyrosine kinase inhibitor, NA-PP1, beginning E16.5 daily for 2 days (dose of 62.5 mg/kg) and kidneys were harvested 4 hours after 2nd injection on E 17.5. Immunoblots were probed with antibodies to Ret or actin as a loading control. Ret antibody recognized both Ret (lower band corresponding to 155 kd) and phopho-Ret (upper band corresponding to 175 kd) and immunoblot revealed kidneys exposed to NA-PP1 had reduced phospho-Ret as well as Ret proteins. Line marks where the membrane was cut for incubation with anti-Ret or anti-actin. **B. Distal colon innervation in Ret$^{flox\text{-}V805A}$ mice exposed to vehicle and NA-PP1.** Pregnant Ret$^{flox\text{-}V805A}$ mice were injected daily with vehicle or a small molecule Ret tyrosine kinase inhibitor, NA-PP1, beginning E15.5 daily for 4 days (top panel, dose of 62.5 mg/kg) or beginning E16.5 for 3 days (bottom panel, dose 50 mg/kg). Colonic innervation pattern was assessed in whole mount distal colons immunostained with neurofilament heavy chain (NFH) to label axonal projections and HuC/HuD to label neuronal cell bodies followed by optical clearing. The pattern of enteric nervous system in the distal colon in mice with exposure to NA-PP1 E15.5 was similar to vehicle-exposed mice (top panel). Medium dose (50 mg/kg) NA-PP1 exposure beginning E16.5 resulted in no difference in enteric neuron cell number in the distal colons compared to vehicle exposure (mean ± SD, vehicle 220 ± 30.7 vs. NA-PP1 205 ± 17.5, Welch’s t-test, p=0.4 vehicle n=5, NA-PP1 n=4).

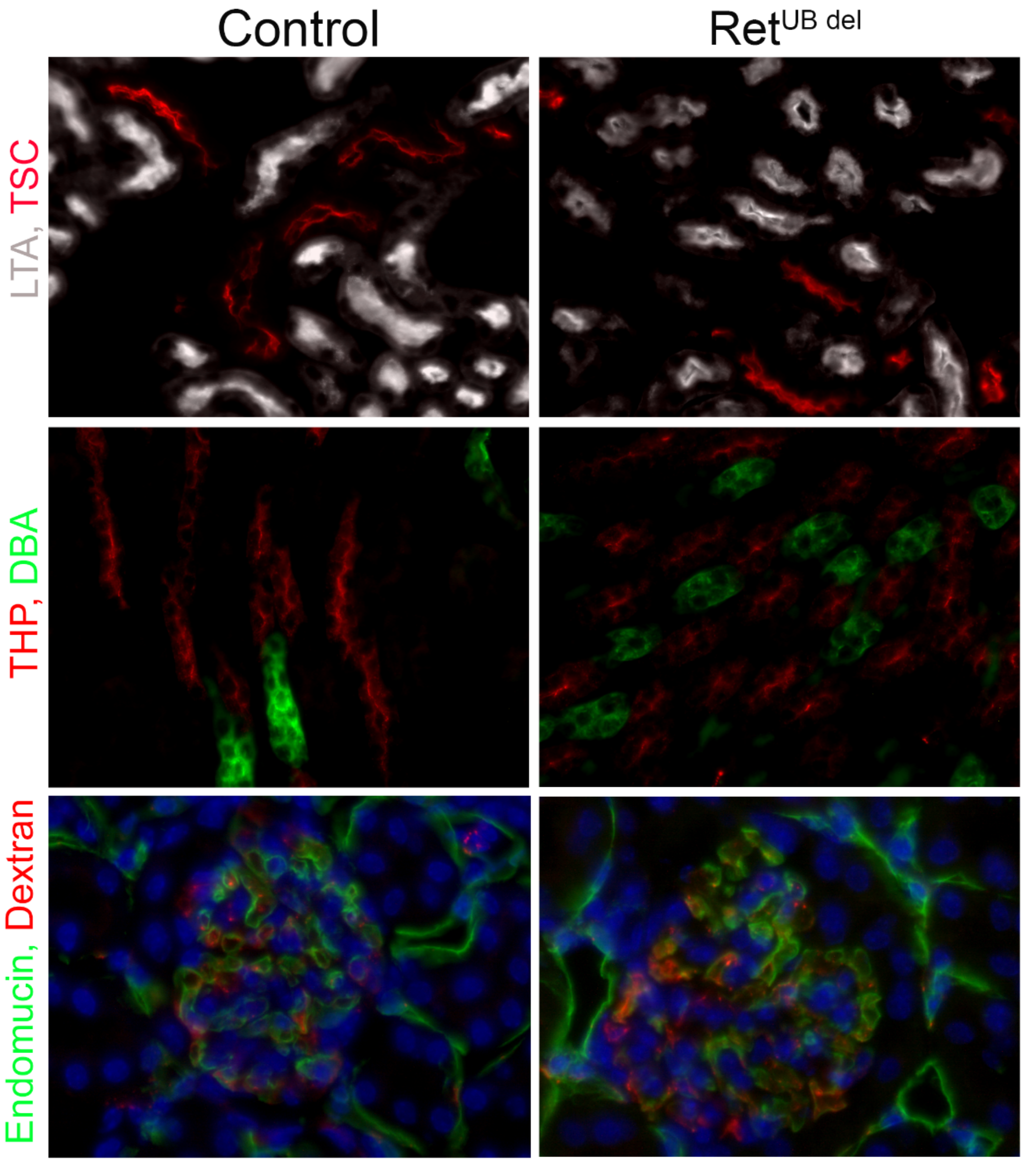

Supplemental Figure 2: Ret$^{UB\ del}$ mice have tubules, glomeruli and capillaries that are similar to controls. Kidneys from mice exposed to Dox beginning E16.5 were analyzed at 2 weeks of age. Kidneys were stained with proximal tubule marker, LTA (white), and distal tubule marker, TSC (red), shown in top panel. Thick ascending limb is labeled with THP (red) and collecting ducts with DBA (green) shown in middle panel. Renal capillaries and small veins are labeled with endomucin (green) in 6 week old mice after injection of high molecular weight dextran (70 kD). High molecular weight dextran is retained in the glomerular capillary loops and peritubular capillaries. While glomeruli appear larger at this age, dextran retention and peritubular vascular density was qualitatively normal in Ret$^{UB\ del}$ mice.

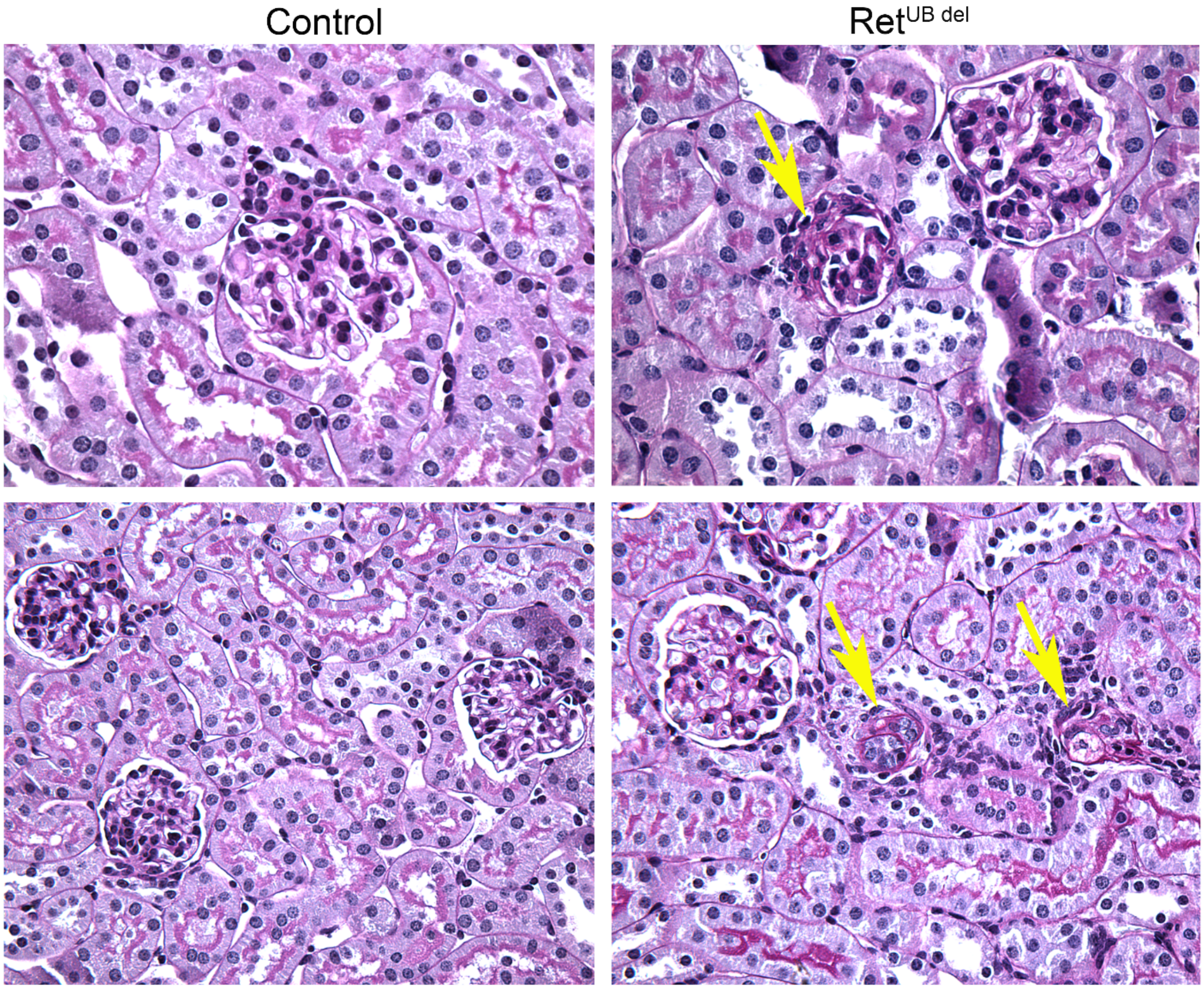

Supplemental Figure 3: Adult Ret$^{UB\ del}$ mice have obsolescent glomeruli and develop pathological changes of CKD. Top, PAS stained kidneys reveal focal obsolescent glomeruli in Ret$^{UB\ del}$ mice, characterized by small, shrunken and partially solidified capillary tufts (arrow) at 12 weeks of age (imaged at 600x). By 12 weeks Ret$^{UB\ del}$ mice develop focal tubular atrophy (arrows) with surrounding areas of interstitial fibrosis and chronic inflammatory cell infiltrates (imaged at 400x).

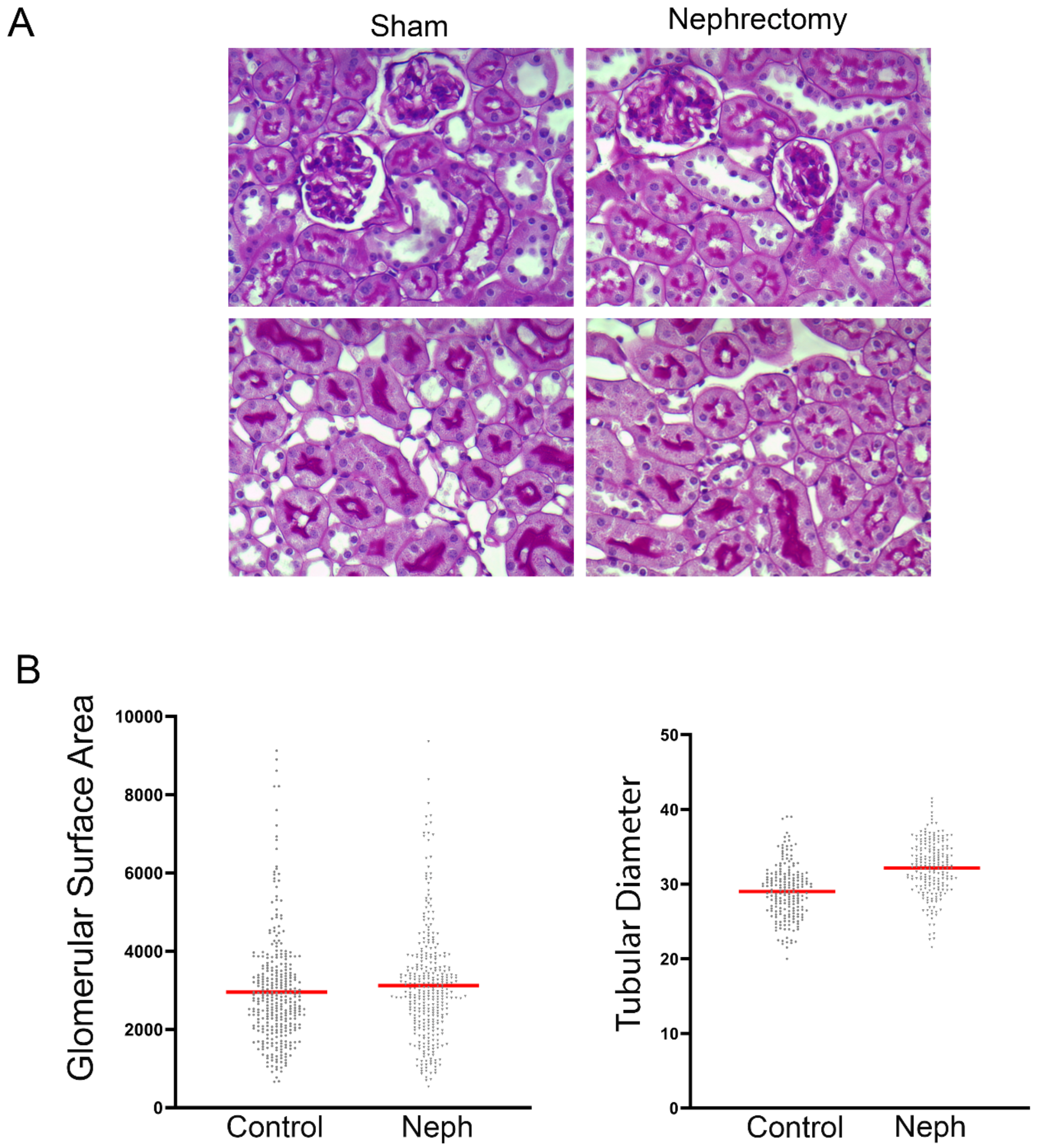

Supplemental Figure 4: Uninephrectomy in adult mice does not cause glomerular hypertrophy but mild proximal tubular enlargement. Eight week old mice with normal nephron endowment ($Ret^{flox\text{-}V805A}$ background) underwent sham operation or uninephrectomy. **A.** PAS stained kidneys 4 weeks after sham or uninephrectomy reveal normal appearing glomeruli and tubules (top panel, imaged at 400x), with no pathologic changes of chronic kidney disease. **B.** Quantification of glomerular surface area and tubular diameter. Glomerular surface area is not significantly increased after nephrectomy (4.3% larger in nephrectomy compared to controls, CI =-4.1, 13.5 p=0.51, n=4 mice per group). In contrast, tubular diameter is 10.8% larger than controls (CI = 0.12, 17.9) or an average of 3.14 µm larger (CI = 1.09, 5.18), p=0.02, n=4 mice per group. Data was analyzed by mixed-effects regression.

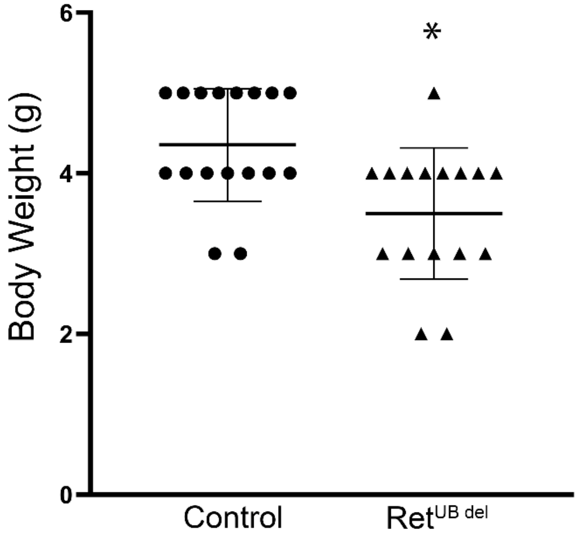

Supplemental Figure 5: Body weight in control and Ret$^{UB\ del}$ mice exposed to gentamicin from P3-9. Pups were weighed on P10, after 7 days of exposure to 100 mg/kg of gentamicin. Ret$^{UB\ del}$ mice had significantly lower body weight compared to controls (mean body weight of 3.5g in Ret$^{UB\ del}$ n=16 compared to 4.3g in controls n=17), *p<0.01, Welch's t-test.

Ret$^{UB\ del}$

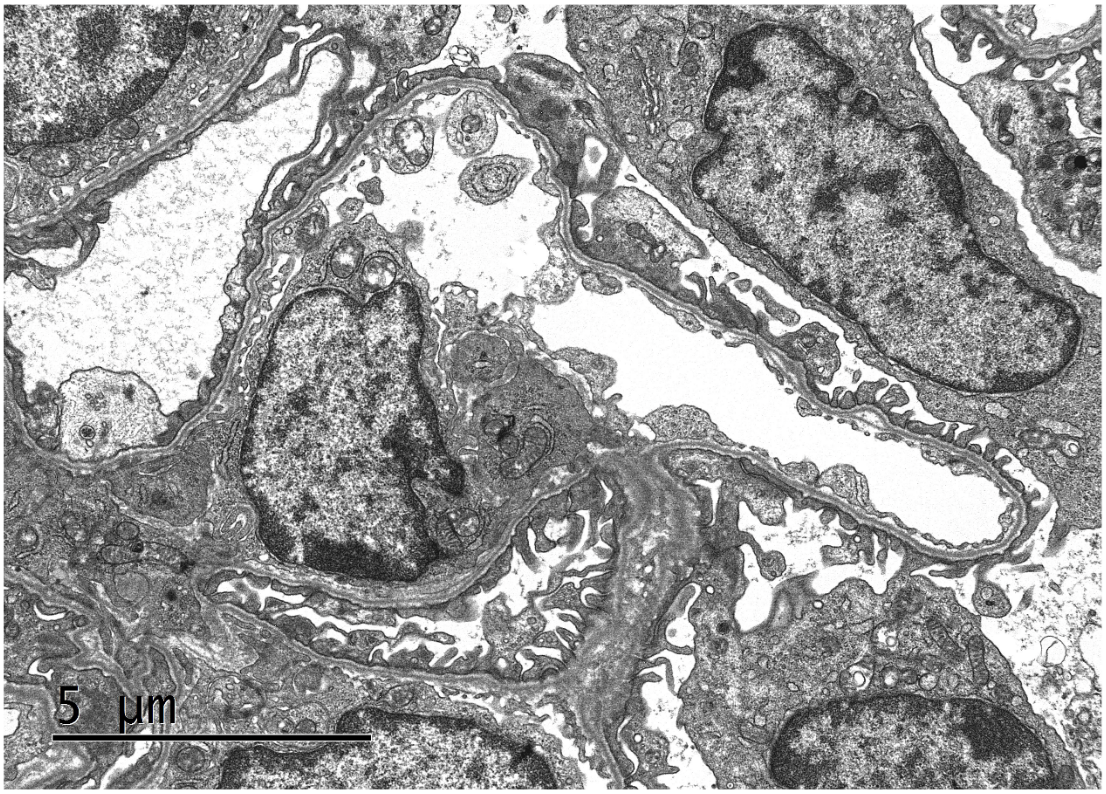

Supplemental Figure 6: Electron micrograph reveal mild focal glomerular injury after gentamicin-induced AKI. While the majority of glomeruli appear intact with minimal pathologic changes, there are areas of short-segment, mild foot process effacement in mice examined 1 day after completion of 7 days of gentamicin injection in a Ret$^{UB\ del}$ mouse.

References

1. Combes AN, et al. An integrated pipeline for the multidimensional analysis of branching morphogenesis. *Nat Protoc* 2014;9(12):2859–2879.

2. Pfaffl MW. A new mathematical model for relative quantification in real-time RT–PCR. *Nucleic Acids Res* 2001;29(9):e45–e45.

3. Rao M, et al. Enteric Glia Regulate Gastrointestinal Motility but Are Not Required for Maintenance of the Epithelium in Mice. *Gastroenterology* 2017;153(4):1068-1081.e7.
